# Tailoring Reconstruction of Co/Cu Mixed Oxide-Derived Tandem Electrocatalysts via *In Situ* Electrochemical Dissolution–Redeposition for Enhanced Nitrate-to-Ammonia Conversion

**DOI:** 10.1021/jacsau.5c01439

**Published:** 2026-01-09

**Authors:** Manuel E. G. Winkler, Rafael G. Yoshimura, Pâmella S. Rodrigues, Matheus P. Sales, Kauan L. Gomes, Itamar T. Neckel, Santiago J. A. Figueroa, João B. Souza, Edson A. Ticianelli, Nirala Singh, Fabio H. B. Lima, Serhiy Cherevko, Raphael Nagao

**Affiliations:** † Institute of Chemistry, 28132 University of Campinas, Campinas, SP 13083-862, Brazil; ‡ Center for Innovation on New Energies, University of Campinas, 13083-084, Campinas, SP, Brazil; § 28334 Forschungszentrum Jülich GmbH, Helmholtz-Institute Erlangen-Nürnberg for Renewable Energy (IET-2), Cauerstr. 1, 91058 Erlangen, Germany; ∥ Brazilian Synchrotron Light Laboratory, 215006 Brazilian Center for Research in Energy and Materials, Campinas, SP 13083-100, Brazil; ⊥ São Carlos Institute of Chemistry, University of São Paulo, São Carlos, SP 13560-970, Brazil; # Brazilian Nanotechnology National Laboratory, Brazilian Center for Research in Energy and Materials, Campinas, SP 13083-100, Brazil; ∇ Department of Chemical Engineering, University of Michigan, Ann Arbor, Michigan 48109-2136, United States

**Keywords:** nitrate reduction, Co/Cu tandem electrocatalyst, reconstruction of Co/Cu mixed oxide-derived electrocatalyst, spectroelectrochemistry

## Abstract

Cobalt- and copper-based oxides have emerged as cost-effective electrocatalysts for the electrochemical nitrate reduction reaction (NO_3_RR) to ammonia. However, the cathodic potentials required for NO_3_RR induce irreversible structural transformations that often compromise catalyst stability and selectivity, depending on the applied electrochemical protocol. To understand the resulting dynamic structure–performance relationships and improve nitrate-to-ammonia conversion, a tandem Co_3_O_4_/Cu_*x*_O electrocatalyst was prepared by electrodeposition followed by thermal treatment, and two surface activation strategies were employed: by cycles of cyclic voltammetry (CV) or by holding at a constant potential by chronoamperometry (CA). The CA-reconstructed Co/Cu mixed oxide-derived electrocatalyst exhibited a higher faradaic efficiency (FE) toward ammonia across the entire potential window studied (0.00 to −0.40 V_RHE_). The reconstruction effects induced by both electrochemical protocols were systematically investigated, revealing morphological, structural, and compositional changes that underpin the improved nitrate-to-ammonia conversion. Furthermore, *in situ* and online electrochemical techniques were employed to identify intermediates and active sites, providing new mechanistic insights into the electrochemical nitrate-to-ammonia conversion pathway. These findings contribute to understanding dynamic reconstruction phenomena and offer design guidelines for more stable and selective mixed oxide electrocatalysts for sustainable ammonia production.

## Graphical abstract

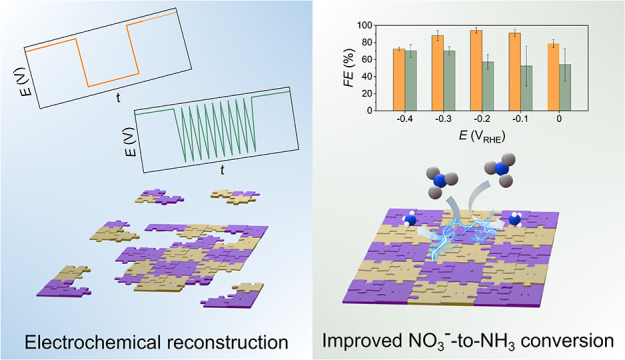

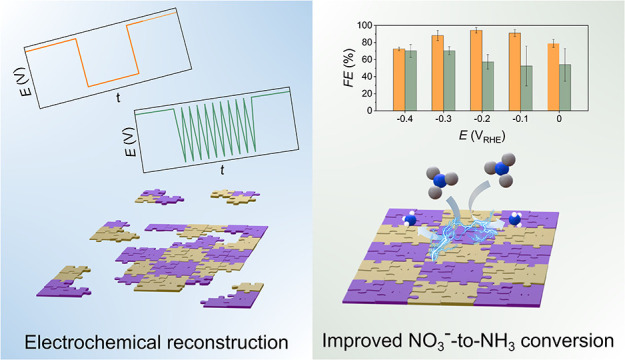

## 1 Introduction

Ammonia (NH_3_) is a crucial chemical in today’s global economy due to its widespread applications as both a fertilizer and a potential carbon-free fuel.^1^ However, the conventional Haber–Bosch process, based on the reaction of N_2_ and H_2_ under high temperature and pressure, remains highly energy-intensive, consuming approximately 2% of the world’s total energy supply and contributing around 1% of global greenhouse gas emissions.^2^ As the demand for ammonia continues to grow, there is an urgent need for more sustainable production routes. In this context, electrochemical nitrate reduction (NO_3_RR) has emerged as a promising green alternative, offering the dual benefits of ammonia synthesis and water remediation, all potentially powered by renewable electricity.^3^

NO_3_RR to ammonia is a complex 8-electron process, which competes with the hydrogen evolution reaction (HER) and produces a series of byproducts, including NO_2_, NO, N_2_, N_2_O, and N_2_H_2_.^4–6^ A variety of electrocatalysts have been designed to improve the activity and selectivity to NH_3_, including transition metal single-atom materials,^7–11^ dual single-atom materials,^12,13^ single-atom alloys,^14,15^ MBenes,^17,18^ and MXenes,^19,20^ among others, but these are limited in large-scale applicability due to their low-yield synthesis, long-term instability, and high cost.^21–23^ Transition metal oxides (TMOs), on the other hand, offer a cost-effective solution with tunable physicochemical properties, such as specific surface area and catalytic activity, which can be tailored by electrocatalyst synthesis methods.^24^ However, TMOs undergo irreversible changes when in contact with the electrolyte and upon polarization at cathodic potentials for NO_3_RR, including phase transitions, morphological changes, and dissolution,^25–31^ which impact their performance. A common practice in TMOs electrocatalysis to minimize degradation or tailor surface reconstruction due contact with the electrolyte (even at open circuit potential) is the application of electrochemical protocols prior to the electrolytic usage, such as cyclic voltammetry (CV),^32^ chronopotentiometry (CP),^33^ or chronoamperometry (CA),^27,28,34^ aiming at surface activation. A series of experimental and theoretical tools have recently been developed to tailor activation, control oxidation, minimize dissolution, and stabilize surfaces, thereby promoting the reliable performance of metal sulfide- and oxide-based electrocatalysts for the oxygen evolution reaction (OER).^33^ However, in most cases, the exact mechanism of activation is poorly understood.

Copper and cobalt metallic phases have been pointed out as promising oxide-derived (OD) electrocatalysts in nitrate-to-ammonia electroreduction.^25,29,35–40^ Studies on the electrocatalytic activity of Cu_2_O nanocubes revealed that an over 90% faradaic efficiency (FE) to ammonia is obtained by the OD-Cu^0^ reconstructed sites with undefined morphology at −0.3 V_RHE_.^41,42^ Spinel Co_3_O_4_, on the other hand, requires more cathodic potential to achieve a similar FE (larger than −0.6 V_RHE_), and the reconstruction includes *in situ* facet transformation from Co_3_O_4_ (112) to (111),^43^ the formation of metallic Co,^44^ and the interconversion of Co^2+^ and Co^3+^ modulated by the applied potential.^45^ Notably, when these oxides are combined at adjacent active sites, forming what is known as a tandem electrocatalyst, they enable a sequential catalytic process, where a reaction intermediate formed at one site is further converted at the neighboring site.^38^ Using this strategy, an FE to NH_3_ of above 90% has been achieved at relatively mild applied potentials (approximately −0.2 V_RHE_) for a Co/Cu tandem electrocatalyst. Additionally, He et al. reported an FE of 93.3% in a core–shell Cu/CuO_*x*_@Co/CoO structure.^25^ Huang et al. developed Cu_2_O@CoO yolk–shell for NO_3_RR and achieved near-to-unity FE, and interestingly, the efficiency above 90% was maintained even at high overpotential (between −0.1 and −0.9 V_RHE_).^37^

Recent studies have highlighted the dynamic reconstruction behavior of Co- and Cu-oxide tandem electrocatalysts during NO_3_RR. *In situ* Raman spectroelectrochemistry revealed that the phase stability of Co and Cu species is highly potential-dependent, with various surface states observed under cathodic conditions, including Cu/CuO_*x*_, Cu–OH_*x*_, CuO_*x*_, CoOH, Co_3_O_4_, Co­(OH)_2_, CoO_*x*_, and metallic Co.^25,29^ Identical-location transmission electron microscopy (IL-TEM) combined with energy-dispersive X-ray spectroscopy (EDX) mapping further demonstrated Cu leaching when the electrocatalyst was subjected to cyclic voltammetry between 0.4 and −0.5 V_RHE_ in a nitrate-containing electrolyte.^29^ Despite the significant progress achieved with Cu- and Co-based oxide tandem systems, several challenges remain, most notably the need for strategies to prevent active site dissolution and maintain long-term catalytic stability.

In this work, we prepared a precatalyst Co_3_O_4_/Cu_*x*_O by electrodeposition and calcination using Cu-foil as the substrate and Cu source. Then, two methods of surface activation were investigated: by CV (10 scan cycles from 0.15 to −0.40 V_RHE_ at 20 mV s^–1^) and by CA (1 h holding at −0.30 V_RHE_) in 1.0 mol L^–1^ NaOH. The performance of the reconstructed samples was evaluated in NO_3_RR, revealing improved FE to NH_3_ by the CA-reconstructed protocol, which was explained by online inductively coupled mass spectrometry (ICP-MS) measurements. Furthermore, the active sites and intermediates were identified by *in situ* X-ray absorption near-edge spectrometry (XANES), *ex situ* X-ray photoelectron spectroscopy (XPS), *in situ* Fourier transform infrared spectroscopy (FTIR), and online differential electrochemical mass spectrometry (DEMS).

## 2 Results and Discussion

### 2.1 Catalyst Design and Characterization

The synthesis route to form the Co_3_O_4_/Cu_*x*_O tandem electrocatalyst consisting of uniformly distributed Co_3_O_4_ nanowires and Cu_*x*_O agglomerates is illustrated in Figure S1. Initially, cobalt was electrodeposited onto a polished Cu-foil from an aqueous solution containing 0.5 mol L^–1^ Co­(CH_3_COO)_2_ and 0.25 mol L^–1^ H_3_BO_3_ at −1.6 V_Ag/AgCl_ for 30 s, exhibiting a uniform nanosheet-like Co layer (Figure S2a) with an average particle size of ∼600 nm (Figure S2b) as well as good surface coverage and adhesion. Subsequently, cobalt oxalate (CoC_2_O_4_) was electrochemically grown in 0.5 mol L^–1^ oxalic acid (H_2_C_2_O_4_) at 0.3 V_Ag/AgCl_ for 30 min, promoting the formation of a nanowire-like morphology with an average diameter of ∼140 nm (Figure S3). The resulting structure was then calcined in air at 400 °C for 2.0 h (heating rate: 15 °C min^–1^). The scanning electron microscopy (SEM) image of the Co_3_O_4_/Cu_*x*_O electrocatalyst (Figure 1a) exhibits two distinct morphologies consisting of a dense nanowire network (∼400 nm diameter) interspersed with spherical porous agglomerates ranging from 1.7 to 3.2 μm. The corresponding SEM-EDX elemental map (Figure 1b) indicates that the nanowires are predominantly cobalt-rich (purple), consistent with Co_3_O_4_, while the agglomerates are enriched in copper (yellow), consistent with Cu_*x*_O formation. Higher-magnification imaging of the Cu-rich region confirms that the Cu_*x*_O agglomerates also exhibit a nanowire-like morphology with a homogeneous Cu distribution (Figure S4). Low-magnification SEM-EDX images (Figure S5a,b) confirm the homogeneous distribution of both components across the electrode surface, including larger Cu_*x*_O agglomerates up to ∼7.7 μm. The corresponding EDX spectrum and quantified elemental compositions are provided in Figure S5c. The edge-positioned SEM micrograph (Figure S6) reveals an electrode thickness of approximately 77 μm, with Co_3_O_4_ nanowires and Cu_*x*_O agglomerates extending to the film edges. This spatially organized tandem architecture suggests distinct functional domains where Co_3_O_4_ nanowires and Cu_*x*_O agglomerates may synergistically contribute to the catalytic performance.

**Figure 1:**
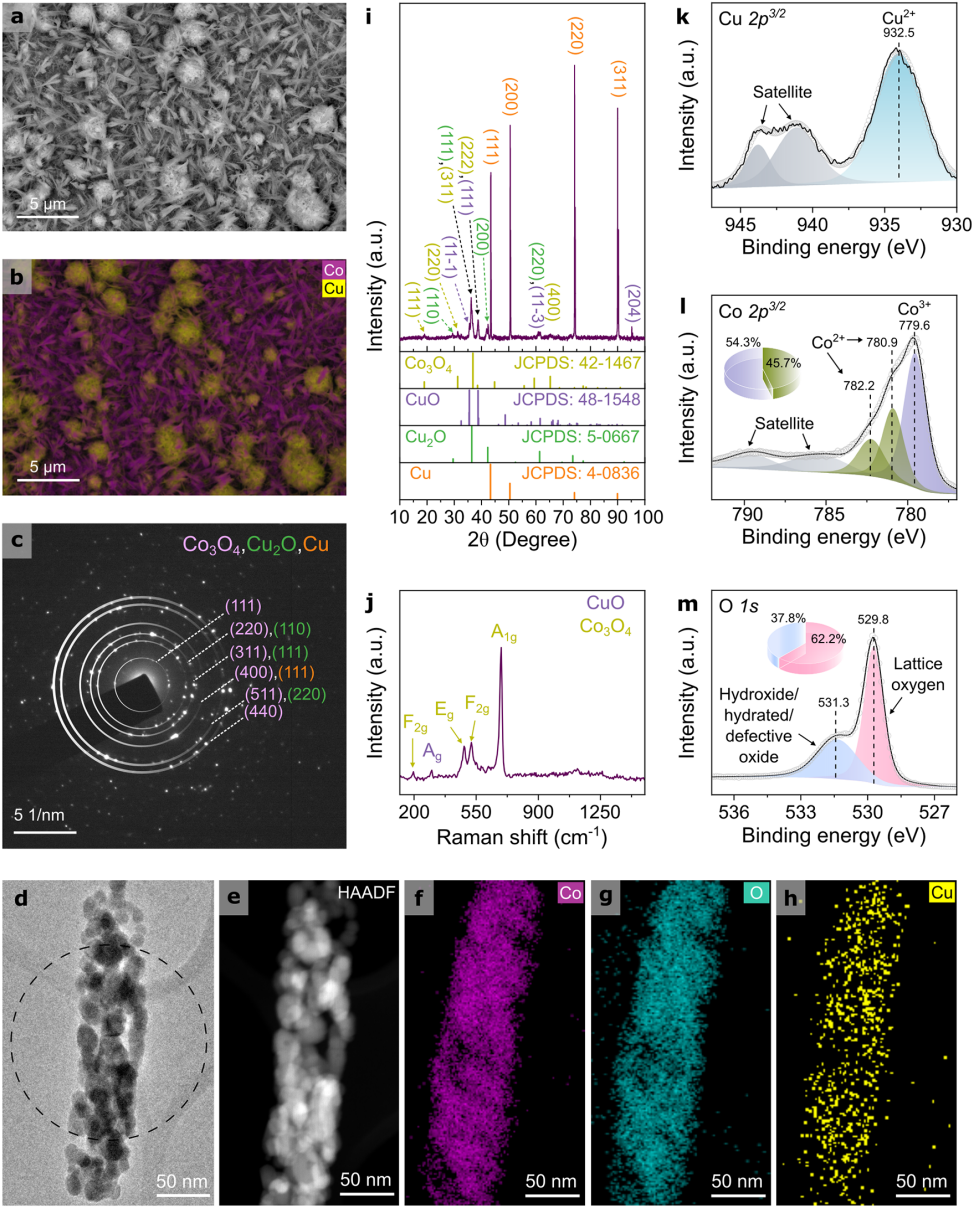
Morphological, structural, and surface characterization of the Co_3_O_4_/Cu_*x*_O precatalyst. (a) SEM image and corresponding (b) SEM-EDX elemental maps of Co (purple) and Cu (yellow). (c) SAED pattern obtained from a single nanowire shown in the (d) TEM micrograph. (e) HAADF-STEM image and corresponding elemental maps of (f) Co, (g) O, and (h) Cu. (i) XRD pattern, (j) Raman spectrum, and high-resolution XPS spectra of (k) Cu 2p^3/2^, (l) Co 2p^3/2^, and (m) O 1s for Co_3_O_4_/Cu_*x*_O.

The chemical analysis of the precatalyst shows that Co is in the Co_3_O_4_ phase, while Cu remains in several oxidation states. The selected area electron diffraction (SAED) pattern (Figure 1c) from a single nanowire (Figure 1d) revealed the polycrystallinity of Co_3_O_4_ with (111), (220), (311), (400), (511), and (440) planes, (110), (111) and (220) planes of Cu_2_O, and Cu (111); *d*-spacing values are provided in Table S1. The attribution of Cu phases was based on the HAADF-STEM micrograph (Figure 1e) and corresponding EDX elemental maps of Co, O, and Cu (Figure 1f–h, respectively), indicating the overlapping of the elements. The XRD pattern (Figure 1i) confirms the presence of cubic Cu_2_O with diffraction peaks at 2θ = 29.45°, 36.18°, 42.41,°, and 61.18°, corresponding to the (110), (111), (200), and (220) planes (JCPDS: 5-0667). Co_3_O_4_ is identified with peaks at 2θ = 19.06°, 31.30°, 36.55°, 38.77°, and 65.38°, assigned to the (111), (220), (311), (222), and (400) planes of the cubic spinel phase (JCPDS: 42-1467). Additionally, monoclinic CuO is detected with peaks at 2θ = 35.59°, 38.77°, 61.18°, and 95.22°, corresponding to the (11–1), (111), (11–3), and (204) planes (JCPDS: 48-1548). Peaks from the underlying polycrystalline Cu-foil are also present at 2θ = 43.44°, 50.54°, 74.20°, and 90.00°, corresponding to the (111), (200), (220), and (311) planes, respectively (JCPDS: 4-0836). The Raman spectrum (Figure 1j) shows the characteristic vibrational modes of Co_3_O_4_, including the F_2g_, E_g_, F_2g_, and A_1g_ modes at 196, 482, 523, and 691 cm^–1^, respectively,^46^ along with the A_g_ mode of CuO at 300 cm^–1^.^47^ XPS analysis (Figure S7) indicates a Co-enriched surface with a Co/Cu atomic ratio of 3.5:1. The Cu 2p^3/2^ peak at 932.5 eV (Figure 1k) confirms the presence of Cu^2+^ species.^42^ The detection of Cu^2+^ species via XPS, alongside the identification of both Cu_2_O (Cu^+^) and CuO (Cu^2+^) phases in XRD, highlights a surface-bulk compositional discrepancy typical of mixed metal oxide systems. This suggests that the surface is predominantly oxidized to CuO, while subsurface or bulk regions retain significant amounts of Cu_2_O. Such a mixed-valence copper environment is of particular interest, as the resulting heterointerface facilitates electron transfer and increases the density of electrochemically active sites.^48^ The Co 2p^3/2^ spectrum (Figure 1l) displays features characteristic of Co_3_O_4_, with contributions from Co^3+^ (779.6 eV, 54.3%) and Co^2+^ (782.2 and 780.9 eV, 45.7%).^49,50^ Moreover, under electrochemical operating conditions, dynamic transformations between these oxidation states are expected, potentially leading to further restructuring or activation of the catalyst surface.^34^ The asymmetric O 1s spectrum (Figure 1m) reveals two main components: (i) lattice oxygen in the metal oxide at 529.8 eV (62.2%) and (ii) hydroxide, hydrated, or defective oxide at 531.3 eV (37.8%).^49,51–54^

### 2.2 Electrochemical Surface Reconstruction and Nitrate Reduction Performance

The electrochemical evaluation of the as-prepared Co_3_O_4_/Cu_*x*_O electrode revealed a dynamic surface reconstruction under reductive conditions. The polarization curves in Figure 2a were collected after three CV cycles in NaOH 1.0 mol L^–1^ (from 0.15 V_RHE_ to −0.40 V_RHE_ at a 1 mV s^–1^ scan rate). Notably, the first two cycles exhibited substantial irreversible features in the current density profiles, characteristic of electrochemical surface reconstruction, suggesting the reduction of surface oxides; from the third cycle onward, the voltammetric response became reproducible. The nitrate-containing (20 mmol L^–1^) linear sweep voltammogram displays a pronounced increase in cathodic current starting at 0.25 V_RHE_ and a multistep reduction pathway, related to NO_3_RR, superposed to three redox processes possibly related to (I) NO_3_^–^ to NO_2_^–^, (II) NO_2_^–^ to NH_3_, and (III) N_2_-related species. Because of the increase in current following surface reconstruction, we hypothesize these transformations are associated with the reduction of surface oxides, leading to the reorganization of the mixed metal oxide interface and the formation of catalytically active sites. As the restructuring appears to stabilize after several CVs, the electrochemical surface area (ECSA) was determined after acquiring reproducible CVs in NaOH by measuring the double-layer capacitance in a nonfaradaic region (Figure S8).

**Figure 2:**
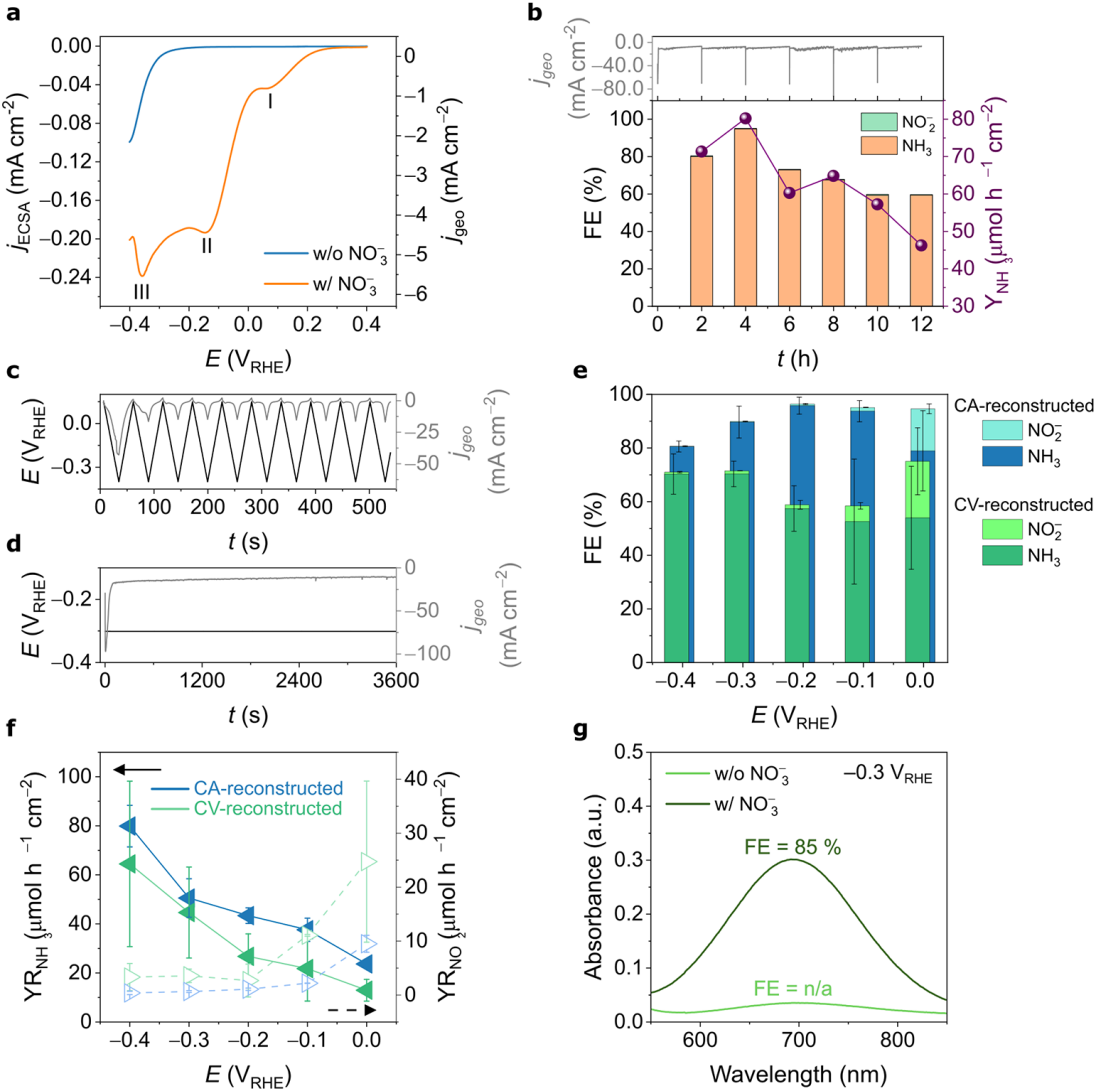
(a) Linear sweep voltammograms (LSVs) of Co/Cu mixed oxide-derived electrocatalyst in 1.0 mol L^–1^ NaOH (blue) and 1.0 mol L^–1^ NaOH + 20 mmol L^–1^ NaNO_3_ (orange). Scan rate: 1 mV s^–1^. (b) Evolution of FE toward NH_3_ and NO_2_^–^ during six consecutive 2 h electrolysis cycles of unactivated Co_3_O_4_/Cu_*x*_O at −0.30 V_RHE_ in 1.0 mol L^–1^ NaOH + 20 mmol L^–1^ NaNO_3_. The electrolyte was refreshed every 2 h. Electrochemical surface reconstruction in 1.0 mol L^–1^ NaOH induced by (c) 10 CV cycles from 0.15 V_RHE_ to −0.40 V_RHE_ (CV-reconstructed) and (d) 1 h chronoamperometry at −0.30 V_RHE_ (CA-reconstructed). (e) Potential-dependent faradaic efficiency (FE) toward NH_3_ and NO_2_^–^ for CA- and CV-reconstructed electrodes. (f) Corresponding NH_3_ and NO_2_^–^ yield rates. (g) Postelectrolysis UV–vis detection of NH_3_ at −0.30 V_RHE_ in nitrate-containing (1.0 mol L^–1^ NaOH + 20 mmol L^–1^ NaNO_3_) and nitrate-free (1.0 mol L^–1^ NaOH) electrolytes.

The performance of nitrate reduction to NH_3_ and NO_2_^–^ under mild conditions (−0.30 V_RHE_) was monitored over six consecutive 2 h electrolysis cycles (Figure 2b). After each cycle, the electrolyte was renewed, and the concentrations of NO_2_^–^ and NH_3_ were quantified by colorimetry (Figures S9 and S10). In the first cycle, the FE to ammonia reached 80.0%, and the maximum was observed in the second cycle, reaching 95.1%, before gradually declining to 59.4% by the sixth cycle, indicating uncontrolled deactivation of the electrocatalyst’s active sites. In the search for improved nitrate-to-ammonia conversion, two controlled activation protocols were established in nitrate-free electrolyte: (i) cyclic voltammetry (CV), where multiple potential cycles drive progressive surface modifications (10 cycles from 0.15 to −0.40 V_RHE_ at 20 mV s^–1^), and (ii) chronoamperometry (CA), performed at a fixed reductive potential of −0.30 V_RHE_ for 1 h, promoting a distinct mode of surface restructuring. Figure 2c,d shows the characteristic current density responses for these activation procedures, highlighting the contrasting surface dynamics induced by potential cycling versus constant potential bias. Additionally, ECSA analysis indicates that both activation protocols increase the number of electrochemically accessible sites, with the CV-reconstructed electrode exhibiting a slightly higher ECSA the CA-treated sample (Figure S11).

The electrode reconstructed using CA exhibited a higher FE toward NH_3_ than that reconstructed by CV. The reconstructed Co/Cu mixed oxide-derived electrodes (hereafter referred to as CA-reconstructed and CV-reconstructed) were evaluated as electrocatalysts for NO_3_RR under 1 h of electrolysis from 0.0 V_RHE_ to −0.40 V_RHE_. As shown in Figure 2e, the CA-reconstructed Co/Cu mixed oxide-derived electrocatalyst exhibited a consistently higher FE toward NH_3_ across the entire potential range compared to the CV-reconstructed one. Specifically, the FE for NH_3_ of the CV-reconstructed catalyst was 54.0 ± 20.2%, 52.6 ± 23.3%, 57.5 ± 8.5%, 70.3 ± 4.8%, and 70.3 ± 7.5% at 0.00, −0.10, −0.20, −0.30, and −0.40 V_RHE_, respectively. In contrast, the CA-reconstructed catalyst achieved markedly higher values of 78.9 ± 14.9%, 93.7 ± 4.0%, 95.8 ± 3.2%, 89.7 ± 5.9%, and 80.6 ± 2.0% at the same potentials. Remarkably, an FE of nearly 96% toward NH_3_ was achieved at −0.20 V_RHE_ for the CA-reconstructed electrode, which is ∼1.7 times higher than that of the CV-reconstructed electrode at the same potential. A comparable FE for the CV-reconstructed material was observed only at a higher nitrate concentration (50 mmol L^–1^, Figure S12).

The product yield rates were also strongly influenced by the reconstruction method (Figure 2f). The CV-reconstructed Co/Cu mixed oxide-derived catalyst consistently exhibited higher NO_2_^–^ yield rates across the potential range, particularly at lower overpotentials (24.73 ± 1.49 and 11.07 ± 0.16 μmol h^–1^ cm^–2^ at 0.0 and −0.1 V_RHE_, respectively, compared to 9.49 ± 1.56 and 2.19 ± 0.02 μmol h^–1^ cm^–2^ for the CA-reconstructed electrode). As the cathodic potential became more negative, the level of NO_2_^–^ was further reduced to NH_3_, reflected by the decreasing NO_2_^–^ yield rate and the concomitant increase in NH_3_ production. Notably, at −0.2 V_RHE_ (the potential delivering the highest FE for NH_3_), the CA-reconstructed electrode achieved an NH_3_ yield rate of 43.37 ± 3.17 μmol h^–1^ cm^–2^, approximately 1.6 times higher than that of the CV-reconstructed electrode (26.68 ± 9.16 μmol h^–1^ cm^–2^). Increasing the nitrate concentration enhanced the NH_3_ yield rate to 138.86 ± 2.04 μmol h^–1^ cm^–2^ for the CA-reconstructed electrode and 91.93 ± 37.19 μmol h^–1^ cm^–2^ for the CV-reconstructed electrode at 0.10 mol L^–1^ (Figure S12). These findings underscore how electrochemical reconstruction critically modulates nitrate reduction pathways, particularly by enhancing selectivity toward ammonia at relatively low overpotentials when conducting CA pretreatment. Co_3_O_4_-based catalysts typically present an FE for NH_3_ of >90% only at more cathodic potentials, −0.6 V_RHE_ or higher,^43,55^ whereas copper oxides achieve this under milder conditions (around −0.3 V_RHE_).^26,42^ Compared to recent studies on Co-, Cu-, and oxide-derived catalysts for NO_3_RR, the CA-reconstructed electrode demonstrates a remarkably high FE for ammonia, especially at −0.20 V_RHE_, where few reports achieve similar selectivity and activity under alkaline conditions, surpassing or matching the values reported for Co/Cu tandem systems.^25,29,37^ It is important to highlight that, unlike conventional nanoparticle-based catalysts, the Co/Cu mixed oxide studied here was prepared by electrodeposition, a synthesis method that inherently produces distinct structural features owing to the strong bonding between the electrocatalyst and substrate.^56^ Such characteristics can strongly influence both the reconstruction process and the catalytic behavior. This suggests that electrodeposition not only offers a scalable and controllable fabrication route but also imparts unique surface and electronic properties that are highly susceptible to tailored electrochemical reconstruction. This capability becomes a powerful lever to optimize electrocatalyst activity and selectivity beyond what is typically achievable with presynthesized nanoparticle-based materials. A performance comparison with literature-reported catalysts is summarized in Table S2.

To confirm that nitrate was the sole nitrogen source for ammonia production, control electrolysis was performed in a nitrate-free electrolyte (1.0 mol L^–1^ NaOH) under conditions identical to those used in the nitrate-containing experiments (−0.30 V_RHE_, 1 h). The UV–vis spectrum of the catholyte, analyzed using the colorimetric reagent for NH_3_ detection, is shown in Figure 2g, with the corresponding chronoamperometric profile provided in Figure S13. No detectable ammonia signal was observed, confirming that ammonia formation does not arise from atmospheric nitrogen-containing contaminants. This result validates the electrochemical setup and unequivocally confirms nitrate as the nitrogen source for the production of ammonia in this system.

### 2.3 Understanding Co_3_O_4_/Cu_*x*_O Reconstruction

Morphological and compositional changes resulting from electrochemical surface reconstruction were investigated via XPS and SEM-EDX analyses. The high-resolution Cu 2p^3/2^ spectra (Figure 3a) show two main components at 932.5 and 934.1 eV, assigned to Cu^0^/Cu^+^ and Cu^2+^ species, respectively. For both samples, the Cu LMM Auger spectra (Figure S14) exhibit a peak maximum at a lower kinetic energy (916.6 eV) compared with that of the pristine precatalyst (917.6 eV), indicating that Cu^+^ is the predominant surface copper species.^51^ Quantitative analysis indicated that the CA-reconstructed surface contained 30.7% Cu^0^/Cu^+^ and 69.3% Cu^2+^, whereas the CV-reconstructed surface exhibited a slightly higher proportion of Cu^0^/Cu^+^ at 46.8% and 53.2% Cu^2+^. The Co 2p^3/2^ experimental envelope (Figure 3b) shows its maximum shifted to a higher binding energy (780.6 eV), reflecting the higher Co^2+^ content in the CA-reconstructed electrocatalyst (88.2% Co^2+^ and 11.8% Co^3+^) compared with that of the CV-reconstructed sample (70.6% Co^2+^ and 29.4% Co^3+^). In addition to the shift in the main peak position, the broader and more intense satellite 2 peak (centered at 786.6 eV) further suggests the presence of Co­(OH)_2_ species on the CA-reconstructed surface.^49^ This interpretation is consistent with the O 1s spectrum, where the most intense peak at 531.3 eV, assigned to hydroxide, hydrated, or defective oxide species, dominates the CA-reconstructed sample (89.7%). In contrast, the CV-reconstructed surface exhibits a higher proportion of lattice oxygen (61.8%), with defective oxide and hydroxyl groups contributing 38.2%.

**Figure 3:**
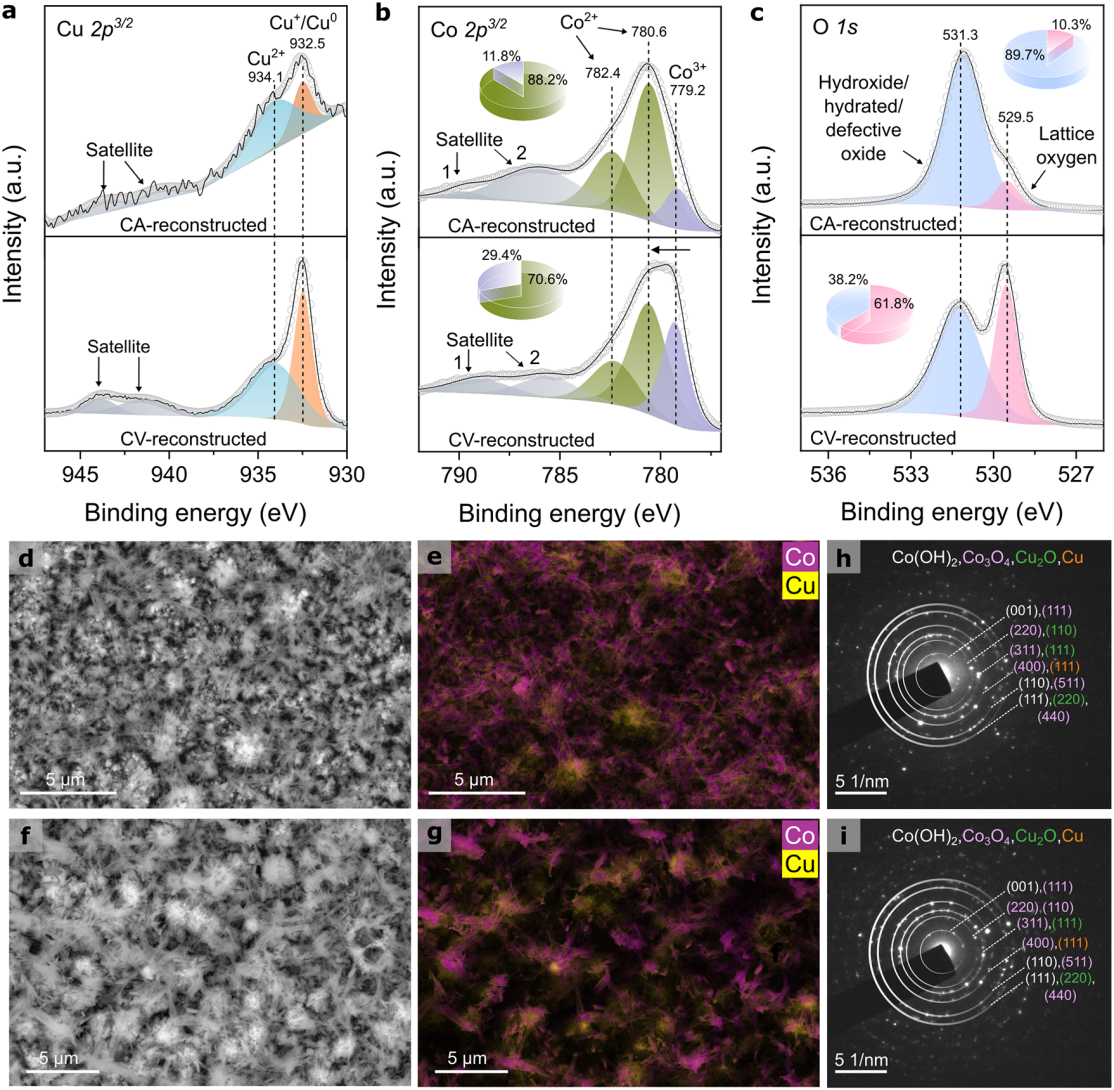
Surface and structural characterization of CA- and CV-reconstructed Co/Cu mixed oxide-derived electrocatalysts. (a) Cu 2p^3/2^ XPS spectra with contributions from Cu^0^/Cu^+^ and Cu^2+^. (b) Co 2p^3/2^ XPS spectra showing the Co^2+^/Co^3+^ distribution. (c) O 1s XPS spectra highlighting differences in hydroxide, hydrated, or defective oxide and lattice oxygen content. (d, e) SEM images and EDX elemental maps of Co and Cu for the CA-reconstructed sample. (f, g) SEM images and EDX elemental maps for the CV-reconstructed sample. Individual maps are shown in Figures S16 and S17. SAED patterns of (h) CA- and (i) CV-reconstructed electrocatalysts.

SEM micrographs reveal distinct differences between the electrodes reconstructed by CA (Figure 3d,e) and CV (Figure 3f,g). The CA-reconstructed sample exhibits thinner nanowires accompanied by surface-deposited particles, suggesting significant surface restructuring. In contrast, the CV-reconstructed sample retains a morphology similar to that of the pristine Co_3_O_4_/Cu_*x*_O material. EDX elemental mapping highlights notable differences in surface composition. The CA-reconstructed electrode presents a cobalt-enriched surface, with Co accounting for 67.4 wt % compared to 38.8 wt % in the CV-reconstructed sample (Figure S15). Conversely, the CV-reconstructed surface is Cu-rich, with a Cu content of 46.8 wt %, significantly higher than the 16.9 wt % observed in the CA-reconstructed electrode. The individual Co and Cu elemental maps are shown in Figures S16 and S17. It is important to note that part of the Cu signal in EDX may originate from the underlying Cu-foil substrate, requiring a more surface-sensitive analysis for further conclusions. XPS was therefore employed to provide more reliable surface composition data. The XPS results confirmed the presence of Co, Cu, O, and C, with Co:Cu atomic ratios clearly reflecting the reconstruction pathway. For the CA-reconstructed electrode, the mass percentages were 53.9% Co, 3.6% Cu, 33.0% C, and 9.5% O, while the CV-reconstructed sample exhibited 47.8% Co, 13.6% Cu, 30.1% C, and 8.6% O (Figure S18). The relatively high oxygen content in both samples is consistent with the presence of oxide and hydroxide species, as confirmed by the high-resolution XPS spectra of Co 2p^3/2^, Cu 2p^3/2^, and O 1s (Figure 3a–c), XRD patterns (Figure S19), STEM-EDX elemental maps (Figures S20 and S21), and SAED patterns (Figure 3h,i).

Structural characterization was performed by XRD and SAED (Figure S19, Figure 3h,i, and Tables S3 and S4), which together confirm the presence of cobalt- and copper-based phases in both CA- and CV-reconstructed samples. In the SAED patterns, the first diffraction ring appears at larger *d*-spacings (4.71 Å for CA-reconstructed sample and 4.75 Å for CV-reconstructed sample) relative to the pristine precatalyst (4.62 Å). Within the resolution of SAED, this ring is consistent with possible overlapping contributions from the Co_3_O_4_ (111) and Co­(OH)_2_ (001) planes, in agreement with the XPS results. Additional rings at 1.60 and 1.50 Å may include Co­(OH)_2_ (110) and (111) contributions, but these cannot be uniquely assigned due to possible overlap with Cu_2_O (220) and Co_3_O_4_ (511) and (440) spacings. SAED further shows reflections attributable to Co_3_O_4_ (311) and Cu_2_O (110) and (111), as well as Cu^0^ (111). XRD additionally reveals Cu_2_O and metallic Cu reflections; although the Cu^0^ peaks cannot be definitively distinguished from substrate contributions, their presence is consistent with the partial Cu reduction observed in the *in situ* XANES measurements discussed in the next section.

It is important to note that the chemical composition and phase distribution observed postreconstruction represent the stable state under *ex situ* conditions. However, under reaction conditions (i.e., constant cathodic potential in an alkaline electrolyte), CV-reconstructed catalysts are likely to evolve toward hydroxide-rich surface states similar to those of the CA-reconstructed sample, given the thermodynamic favorability of Co­(OH)_2_ formation at reducing potentials in alkaline media.^57^ Therefore, the distinct catalytic performances cannot be solely attributed to the presence of Co­(OH)_2_ but also reflect intrinsic differences in morphology, defect structure, Cu dispersion, and the nature of the oxide/hydroxide interfaces introduced during the reconstruction process. These factors collectively influence the nitrate reduction performance.

### 2.4 *In Situ* Identification and Spatial Distribution of Active Sites

Owing to the heterogeneous nature of the Co_3_O_4_/Cu_*x*_O electrocatalyst, a 20 × 20 μm^2^ area was analyzed by *in situ* spatially resolved Co and Cu K-edge XANES. The area of interest was selected based on the *in situ* X-ray fluorescence (XRF) distribution of the Co and Cu elements (Figure S22) acquired in 1.0 mol L^–1^ NaOH + 20 mmol L^–1^ NaNO_3_ at open circuit potential (OCP). The XANES spectra were then collected at OCP and at the optimum nitrate reduction potential (−0.20 V_RHE_) after CA-reconstruction (Figure 4). Principal component analysis (PCA) was applied to reconstruct the spectra at each pixel from the stack of X-ray fluorescence (XRF) images collected across the Cu and Co K-edge XANES spectra.^58^ Single-point analysis was also conducted at the center of the analyzed area (beam size of 30 nm, Figures S23 and S24).

**Figure 4:**
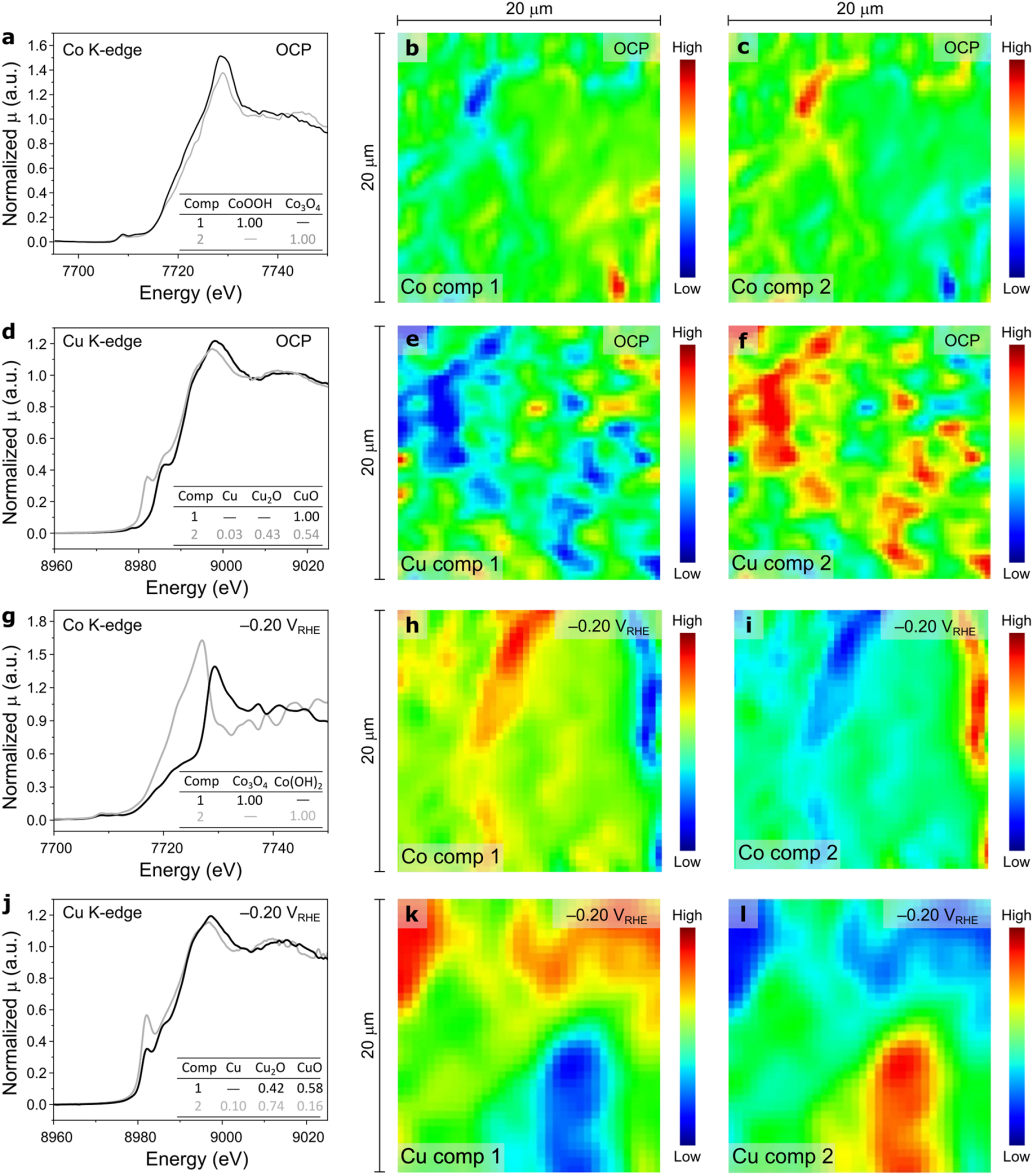
*In situ* spatially resolved distribution of CoOOH, Co_3_O_4_, CuO, Cu_2_O, and Cu species of a 20 × 20 μm^2^ (pixel of 400 nm) catalyst area using linear combination analysis (LCA) through a stack of synchrotron X-ray fluorescence (SXRF) images acquired over the Co and Cu K-edge XANES spectra at (a–f) unactivated Co_3_O_4_/Cu_*x*_O at open circuit potential and (g–l) CA-reconstructed Co/Cu mixed oxide-derived electrocatalyst at −0.20 V_RHE_ in 1.0 mol L^–1^ NaOH + 20 mmol L^–1^ NaNO_3_.

The K-edge XANES spectra of both Co and Cu display two distinct regions: the pre-edge, located at lower energies, which is highly influenced by the low symmetry and coordination number around the absorbing atoms, and the white-line peak, the most intense feature, corresponding to dipole-allowed transitions from 1s to 4p or continuum states.^59–61^ The pre-edge is usually assigned to 1s to 3d transitions, quadrupole transition, which are dipole-forbidden but may gain intensity through p–d orbital mixing, typically in low coordination or distorted environments.^59,61^ At the OCP, the Co K-edge XANES map exhibits spectra consistent with the presence of two components: Comp 1, attributed to Co_3_O_4_, and Comp 2, attributed to CoOOH (Figure 4a). The corresponding spatial distribution maps (Figure 4b,c) reveal the presence of Co_3_O_4_- and CoOOH-rich regions. Additionally, the green regions correspond to the intermediate intensities of both Co_3_O_4_ and CoOOH components, suggesting the presence of mixed-phase domains overlaid. According to the Co Pourbaix diagram (Figure S25), within the pH of this study (pH = 12), the formation of the CoOOH phase is expected at oxidizing potentials (higher than ∼1.1 V_RHE_). Nevertheless, its occurrence has been reported outside this potential range and through phase transformation from Co_3_O_4_ and/or CoO in alkaline media.^62,63^ For copper, the Cu K-edge XANES reveals a more heterogeneous redox state, corresponding to two distinct components: Comp 1, composed of CuO, and Comp 2, consisting of a mixture of Cu^2+^ (0.54), Cu^+^ (0.43), and Cu^0^ (0.03) (Figure 4d). The spatial maps indicate that CuO is relatively segregated from regions containing more reduced Cu species, suggesting a heterogeneous distribution of copper oxidation states over the catalyst surface (Figure 4e,f).

At the potential of maximum FE (−0.20 V_RHE_), two spectra were extracted from the Co K-edge XANES map (Figure 4g). It should be noted that both spectra exhibited deformations that could not be fitted by standard references. Hence, the K-edge position of each extracted component was determined from the maximum of the first derivative curve. The K-edge position of Comp 1 (7727.5 eV) matches that of the Co_3_O_4_ standard (Figure S23b), indicating the stability and preservation of the spinel structure, consistent with its larger spatial area in the map (Figure 4h). In contrast, Comp 2 displayed a K-edge position at 7721.8 eV, close to the Co­(OH)_2_ standard, suggesting cobalt atoms with an oxidation state near +2 with an intermediate intensity covering almost the entire analyzed area (Figure 4i, Figure S23b). Furthermore, as expected, the application of reductive potentials led to the conversion of CoOOH species into more reduced forms of cobalt. From the Cu K-edge XANES maps at −0.20 V_RHE_, two mixed composition components were extracted: Comp 1, Cu^2+^ (0.58) and Cu^+^ (0.42); and Comp 2, Cu^2+^ (0.16), Cu^+^ (0.74), and Cu^0^ (0.10) (Figure 4j). The results indicate partial reduction of the CuO phase to the predominant Cu_2_O sites, consistent with the *ex situ* characterization.

Additional Co and Cu K-edge XANES measurements were performed at the center of the previously analyzed region. The Co K-edge XANES spectra are consistent with the Co_3_O_4_ standard (Figure S23a). However, changes were observed in the pre-edge region as the potential was varied from OCP to −0.2 V_RHE_, showing a stable energy position accompanied by a decrease in intensity (Figure S23a, inset). We interpret this decrease as indicative of an increased occupancy of 4d–3d hybridized orbitals and/or an increase in local symmetry around the absorbing atom under the applied reductive potential. From the localized *in situ* Cu K-edge XANES spectra (Figure S24), linear combination analysis^64^ was employed to extract compositional data, as summarized in Table S5. At the OCP, CuO was identified as the dominant phase. Upon applying more reductive potentials, a change in phase composition was observed, with metallic Cu reaching 44% at −0.20 V_RHE_. Minor differences between the localized XANES spectra and the XANES maps can be attributed to the integration time per point and per pixel (500 ms for the single-point analysis and 8 ms for the 2D XANES). Consequently, point-XANES has a better signal-to-noise ratio (SNR) and higher resolution in the spectrum features. In contrast, the spatially resolved XANES mapping provides a broader and more representative overview of the catalyst oxidation states across a larger analyzed area (20 × 20 μm^2^). This distinction highlights the importance of combining both techniques to better understand the spatial heterogeneity in the material.

### 2.5 Identification of Reaction Intermediates and Mechanistic Understanding

*In situ* Fourier transform infrared spectroscopy (FTIR) and online differential mass spectrometry (DEMS) were performed to elucidate the NO_3_RR reaction pathway on the CA-reconstructed electrocatalyst. The *in situ* FTIR spectra (Figure 5a) revealed characteristic vibrational bands associated with the NO_*x*_ species and hydrogenated intermediates. The band at 1361 cm^–1^ is attributed to NO_2_^–^ and NO species,^65,66^ while the bands at 1450 and 1542 cm^–1^ are assigned to N–H^67–69^ and H–O–N^70,71^ bending, respectively, indicating hydrogenated nitrogen intermediates. These signals arise as early as 0.10 V_RHE_. Additionally, the δ­(H–O–N) vibration appears at −0.1 V_RHE_, suggesting that ammonia formation proceeds via the hydrogenation of NO intermediates. DEMS analysis provided complementary insights into volatile and gaseous intermediates and products. Detected ionic fragments include H_2_^+^ (*m*/*z* = 2), NH_3_^+^ (*m*/*z* = 17), N_2_^+^ (*m*/*z* = 28), NO^+^ (*m*/*z* = 30), N_2_H_2_^+^ (*m*/*z* = 30), N_2_H_4_^+^ (*m*/*z* = 32), and N_2_O^+^ (*m*/*z* = 44). It is noted that *m*/*z* = 17 may also contain contributions from OH^+^ due to water fragmentation.^72^ The signal at *m*/*z* = 33, characteristic of hydroxylamine (NH_2_OH^+^), was not detected, indicating the absence of hydroxylamine as an intermediate under the tested conditions. This conclusion further supports the attribution of *m*/*z* = 32 exclusively to N_2_H_4_^+^, validated by the absence of *m*/*z* = 33. The DEMS profiles (Figure 5b) show that H_2_, NH_3_, and NO are produced from −0.20 to −1.00 V_RHE_, while N_2_-related species (N_2_^+^, N_2_H^+^, N_2_H_2_^+^, N_2_H_4_^+^, and N_2_O^+^) emerge from −0.40 V_RHE_ onward. Consequently, the *m*/*z* = 28 signal reflects contributions from N_2_ and other N_2_-based fragments, while the *m*/*z* = 30 signal corresponds to NO formation from −0.20 to −0.40 V_RHE_ and to N_2_H^+^ (hydrazine fragment) at more negative potentials. A key observation is the absence of hydroxylamine (*m*/*z* = 33), even at high overpotentials (−1.00 V_RHE_), indicating that the typical pathway involving hydroxylamine hydrogenation to ammonia, frequently reported for Cu-based catalysts,^42,66,73–75^ might not occur in the Co/Cu mixed oxide-derived electrocatalyst or that hydroxylamine, if formed, is too short-lived to accumulate to detectable levels. Furthermore, hydrazine (N_2_H_4_) was identified as a byproduct rather than an intermediate, as confirmed by control experiments showing no ammonia production from N_2_H_4_ electroreduction (Figure S27). These findings suggest that N–N coupling occurs via the formation of N_2_O (*m*/*z* = 44) as an intermediate, which subsequently undergoes hydrogenation to form hydrazine, as proposed by de Vooys et al.^5^

**Figure 5:**
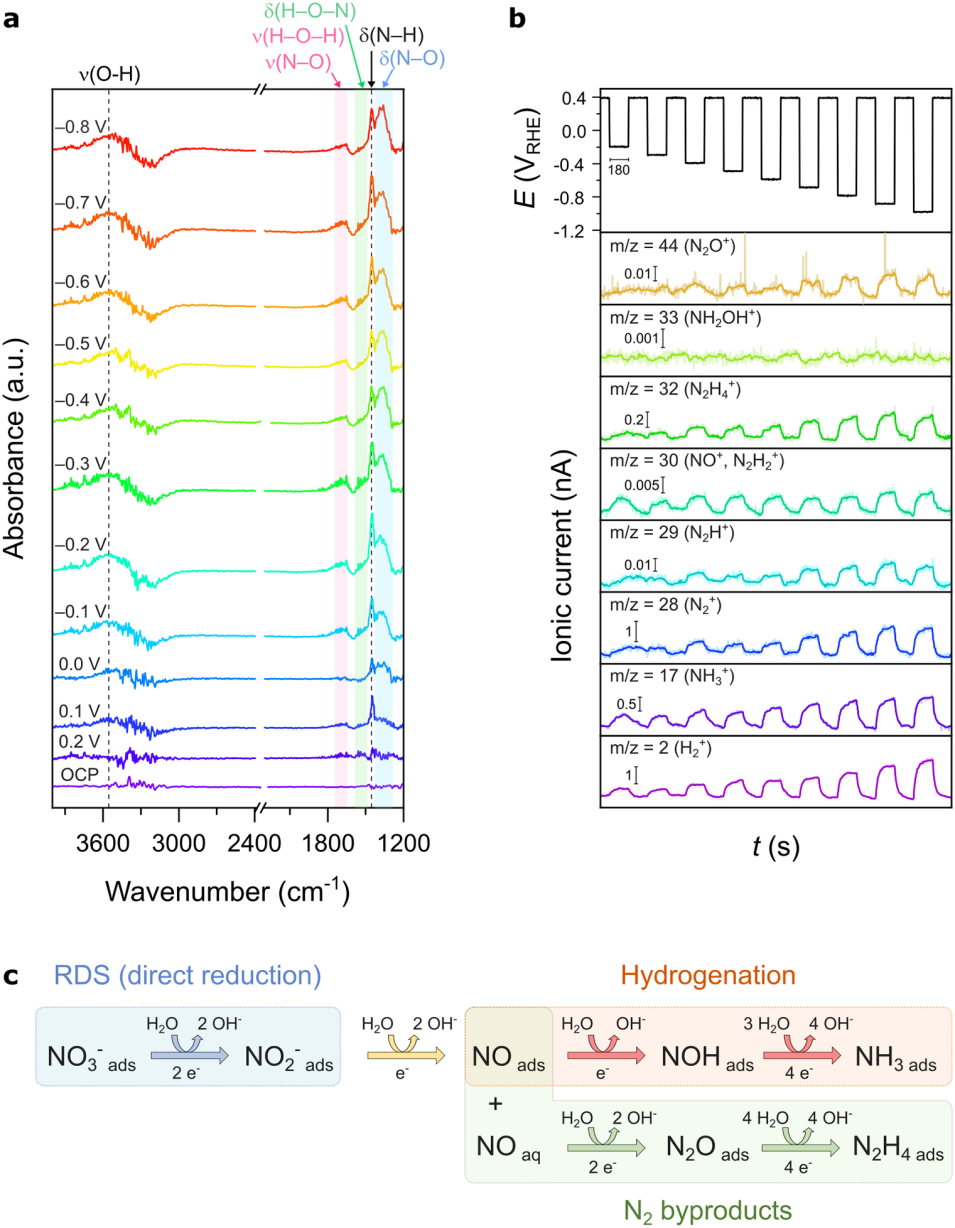
(a) *In situ* FTIR spectra in 0.01 mol L^–1^ NaOH and 0.05 mol L^–1^ NaNO_3_, shown with vertical offset for clarity. (b) Online DEMS spectra acquired in 1.0 mol L^–1^ NaOH and 0.05 mol L^–1^ NaNO_3_. The applied potential versus time is represented at the top, followed by the ionic current of possible ionic fragments present in the reaction (*m*/*z* = 2, 17, 28, 29, 30, 32, 33, and 44). (c) Proposed mechanism based on the intermediates detected.

Based on the combined evidence from *in situ* FTIR and online DEMS, along with electrochemical analysis, a comprehensive reaction pathway for nitrate reduction on the CA-reconstructed Co/Cu mixed oxide-derived electrocatalyst is proposed. As depicted in Figure 5c, the reaction initiates with the reduction of NO_3_^–^ to NO_2_^–^ via a two-electron transfer step, which is the rate-determining step (RDS) of the NO_3_RR.^76^ Subsequently, NO_2_^–^ undergoes a one-electron reduction to form NO, a pivotal intermediate that serves as a branching point toward multiple product pathways.^2,77^ Two main competitive routes are identified for NO. In the first, sequential hydrogenation of NO leads to the formation of NH_3_, which represents the desired product. In parallel, a competing pathway involves N–N coupling to form N_2_-related byproducts. This pathway proceeds through the formation of N_2_O, likely via a two-electron coupling reaction between an adsorbed NO species (NO_ads_) and a NO molecule in solution (NO_aq_), as reported previously.^5^ Subsequent hydrogenation of N_2_O results in the formation of hydrazine (N_2_H_4_), which is detected as a byproduct but does not act as an intermediate toward NH_3_ formation.

These mechanistic insights can be directly correlated to the potential-dependent evolution of the active catalyst phases. Electrocatalytic performance is highly influenced by the dynamic formation of Co^*x*+^ and Cu^*y*+^ species in the cathodic regime of NO_3_RR.^25^ The high selectivity toward NH_3_ at low overpotentials (−0.20 V_RHE_) is attributed to the cooperative action of Cu^+^ sites,^78^ which favor NO_3_^–^ to NO_2_^–^ reduction,^26^ followed by further hydrogenation to NH_3_ at Co^2+^ sites.^25^ However, as the potential becomes more negative (beyond −0.40 V_RHE_), more reduced Co and Cu species become active, potentially promoting N–N coupling pathways that lead to N_2_-related byproducts. This synergy between highly reduced Co and Cu sites can shift the selectivity away from NH_3_ under harsher cathodic conditions. These findings offer critical guidance for the rational design of future NO_3_RR catalysts, highlighting the importance of stabilizing optimal oxidation states to suppress undesirable side reactions. In this context, future theoretical simulations, particularly those capable of capturing the dynamic phase and morphology evolution, would be valuable for testing key mechanistic hypotheses and further refining the understanding of nitrate reduction on mixed oxide systems.

### 2.6 Dissolution Dynamics during Reconstruction and Catalysis

A comprehensive understanding of dissolution during pretreatment and nitrate reduction is fundamental to improving the stability and overall performance of Co/Cu mixed oxide-derived electrocatalysts. To this end, the real-time dissolution of Co and Cu was monitored using a scanning flow cell (SFC) coupled with online inductively coupled plasma mass spectrometry (ICP-MS) during CV- and CA-induced reconstruction (Figure 6a,b) as well as under NO_3_RR conditions. Under the alkaline conditions (pH = 12.3) and within the applied potential window, soluble species are thermodynamically predicted: HCoO_2_^–^ for Co and HCuO_2_^–^ (at *E* ≥ 0.28 V_RHE_) or Cu^+^ (at *E* ≤ 0.28 V_RHE_) for Cu (Figures S25 and S26, in which 0.28 V_RHE_ corresponds to −0.447 V_SHE_ at pH = 12.3). Experimentally, both metals dissolve transiently during oxide reduction with the onset of dissolution at −0.30 V_RHE_ for Co and 0.67 V_RHE_ for Cu (Figure 6c). Consequently, as the CV protocol extends to −0.40 V_RHE_, the transient dissolution of Co becomes pronounced, whereas in CA at −0.30 V_RHE_, Cu dissolution dominates. Notably, Cu dissolution precedes the potential range relevant to the NO_3_RR, suggesting dynamic surface processes involving both dissolution and possible redeposition occurring concurrently with electrocatalysis of the NO_3_RR. In contrast, the onset of Co dissolution occurs at −0.30 V_RHE_, within the NO_3_RR operating window, suggesting that Co remains relatively stable under typical NO_3_RR conditions but may undergo reductive dissolution when pushed toward more negative potentials. Such transient dynamics have been recently pointed by Yoon et al.,^79^ who employed *ex situ* ICP-MS and *in situ* STEM and XANES to track Cu_2_O reconstruction in neutral NO_3_RR, and by Zhang et al.,^29^ who used identical-location TEM-EDX to monitor single-entity Cu_2_O and Co_3_O_4_ nanoparticles in alkaline NO_3_RR.

**Figure 6:**
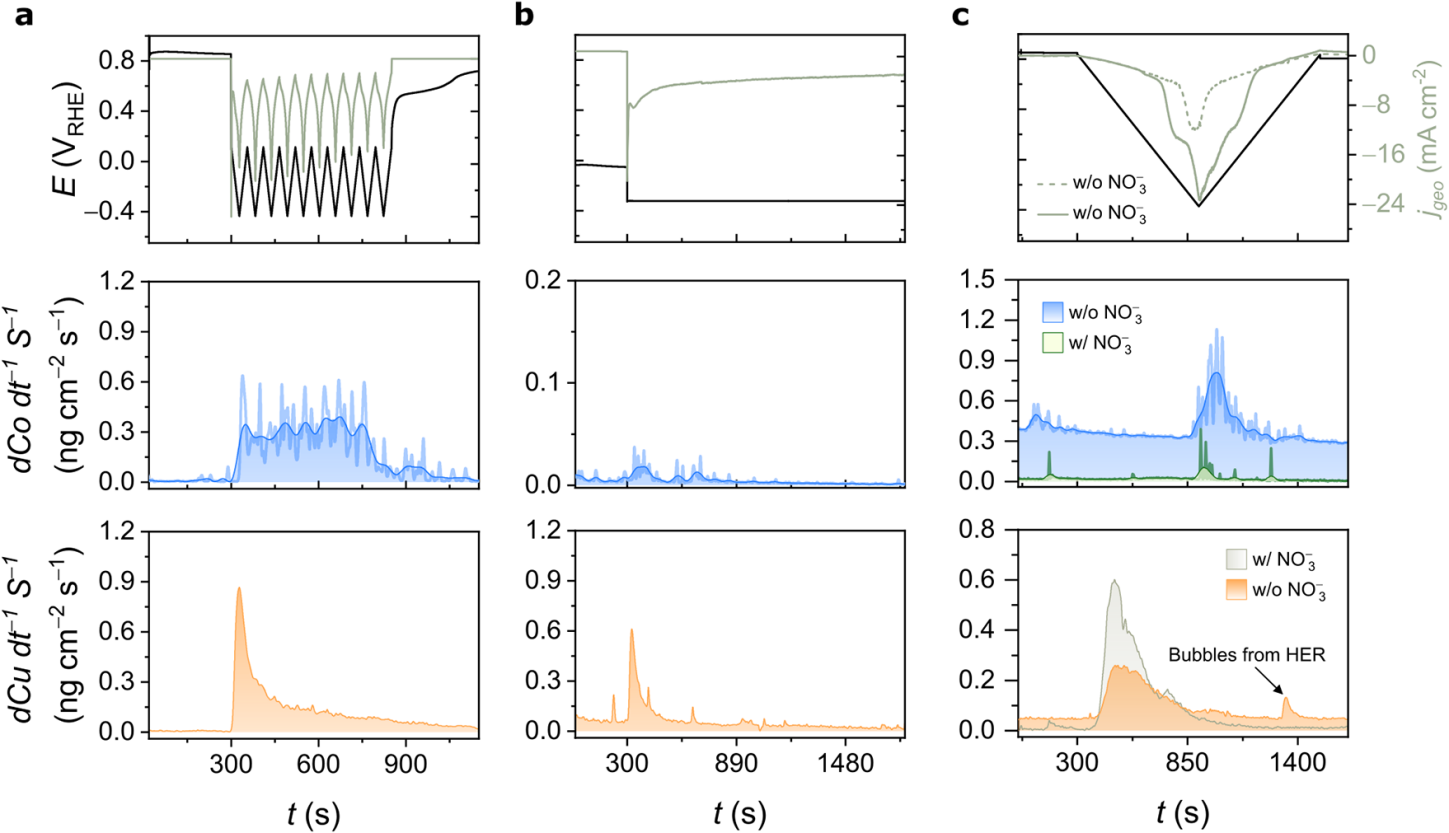
Real-time dissolution profiles of Co and Cu during the (a) CV- and (b) CA-reconstruction protocols in nitrate-free electrolyte (w/o NO_3_^–^: 0.02 mol L^–1^ KOH). (c) Dissolution profile during CV (from 0.80 to −0.40 V_RHE_ at a 2 mV s^–1^ scan rate) in nitrate-containing electrolyte (w/ NO_3_^–^: 0.02 mol L^–1^ KOH + 0.02 mol L^–1^ KNO_3_) and nitrate-free electrolyte (w/o NO_3_^–^: 0.02 mol L^–1^ KOH). Co dissolution curves were denoised using a fast Fourier transform filter, and the original data are presented behind the smoothed curves. Note that all of the current densities are plotted on the same scale.

ICP-MS data align with the elemental quantification obtained from SEM-EDX mapping, comparing the composition of the pristine electrode with that after one CV cycle at similar conditions, both in the presence and absence of nitrate (Figures S28 and S29). The results obtained by SEM-EDX indicate that cobalt loss is approximately six times higher in the alkaline medium without nitrate. Cu, on the other hand, showed an increase in weight percentage, which can be attributed to the exposure of the Cu-foil substrate. In addition, the microstructural evolution induced by the NO_3_RR conditions is shown in Figure S30, revealing a complex surface composed of hexagonal particles, nanowire-like structures, and nanosheet-like features.

## 3 Conclusions

Mastering electrocatalyst reconstruction is challenging for developing efficient nitrate-to-ammonia technologies. Precatalyst Co_3_O_4_/Cu_*x*_O was fully characterized before and after reconstruction in 1.0 mol L^–1^ NaOH by (i) 10 CV cycles from 0.15 to −0.40 V_RHE_ and (ii) 1 h chronoamperometry at −0.30 V_RHE_. Postreconstruction characterization revealed that the precatalyst Co_3_O_4_/Cu_*x*_O undergoes morphological, surface, and structural reconstruction due to the cathodic potential. This restructuring can be tailored by the electrolyte composition and electrochemical protocol (CA or CV) to improve the overall NO_3_^–^-to-NH_3_ performance. CA-reconstruction offers a straightforward approach, minimizing the Co dissolution, as seen in time-resolved dissolution data obtained by SFC-ICP-MS. 96% FE to NH_3_ was obtained at −0.20 V_RHE_ using the CA-reconstructed Co/Cu mixed oxide-derived electrocatalyst, a condition in which Cu^+^ converts NO_3_^–^ to NO_2_^–^ and Co^2+^ adjacent sites further reduce to ammonia by consecutive hydrogenation, as revealed by the *in situ* FTIR and online DEMS experiments. At higher cathodic potential (−0.40 V_RHE_), the FE is diminished by the rise of parallel reactions, such as N_2_ byproducts (N_2_H_4_) possibly facilitated by more reduced Co and Cu sites. These findings provide an indispensable comprehension of the dynamics of electrochemical reconstruction on Co- and Cu-oxide-based electrocatalysts in alkaline electrolysis.

## 4 Experimental Section

### 4.1 Chemicals

Cobalt­(II) acetate tetrahydrate (Co­(CH_3_COO)_2_·4H_2_O, Sigma-Aldrich, ≥98.0%), boric acid (H_3_BO_3_, Sigma-Aldrich, 99.8%) oxalic acid (H_2_C_2_O_4_, Sigma-Aldrich, ≥98.4%), sodium hydroxide (NaOH, Sigma-Aldrich, 99.6%), sodium nitrate (NaNO_3_, Sigma-Aldrich, 99.9%), sulfanilamide (H_2_NC_6_H_4_SO_2_NH_2_, Sigma-Aldrich, 99.6%), N-(1-naphthyl)­ethylenediamine dihydrochloride (C_12_H_16_Cl_2_N_2_, Sigma-Aldrich, 99%), phosphoric acid (H_3_PO_4_, Sigma-Aldrich, ACS reagent ≥ 85%), potassium hydroxide (KOH, Sigma-Aldrich, 99.99%), and potassium nitrate (KNO_3_, Sigma-Aldrich, 99.995%) were used as received. High-purity gases were used for sparging the electrochemical cells, nitrogen (White Martins, 99.999%) and argon (White Martins, 99.999%). Cu-foil (99.99%) served as the substrate to obtain Co_3_O_4_/Cu_*x*_O. All solutions were prepared using high-purity Milli-Q water (18.2 MΩ cm).

### 4.2 Obtaining the Co_3_O_4_/Cu_*x*_O Electrocatalyst

Co_3_O_4_/Cu_*x*_O was obtained by electrodeposition of Co on a polished Cu-foil (1.0 cm × 1.0 cm × 0.1 mm) at −1.6 V_Ag/AgCl_ in 0.5 mol L^–1^ Co­(CH_3_COO)_2_ and 0.25 mol L^–1^ H_3_BO_3_ for 30 s. Then, by applying 0.3 V_Ag/AgCl_ for 30 min in 0.5 mol L^–1^ H_2_C_2_O_4_, cobalt oxalate (CoC_2_O_4_) was electrochemically grown on the electrode surface. Finally, the obtained sample was calcinated in air atmosphere at 400 °C for 2 h (heating rate: 15 °C min^–1^) in a tubular furnace. All electrochemical procedures were conducted in a conventional four-electrode cell with fixed position, using Ag/AgCl_KCl(sat)_ as the reference electrode, two Pt foil (1.0 cm × 1.0 cm × 0.1 mm) connected as the counter electrodes, and Cu-foil as the working electrode, positioned at the center of the two Pt electrodes, controlled by a Metrohm Autolab PGSTAT402N instrument.

### 4.3 Physical Characterization

Scanning electron microscopy (SEM) images and energy-dispersive X-ray (EDX) elemental maps were performed in a FEI Quanta 250 electron microscope, operating at 20 kV. Raman spectra were collected in a Witec Alpha 300 micro-Raman confocal microscope using a 532 nm excitation laser. Surface compositions were assessed by X-ray photoelectron spectroscopy (XPS) in a Thermo Scientific K-alpha spectrometer (Al Kα gun source, 300 μm spot size, 50.0 eV pass energy, and 0.100 eV step size). XRD patterns were obtained in a Bruker D8 Advance Eco diffractometer (Cu Kα, λ = 1.5406 Å) from 2θ = 10 to 100° at a 0.2° step size and were background subtracted using HighScore Plus v3.0.0. Transmission electron microscopy (TEM) images, corresponding EDX elemental maps, and selected area electron diffraction (SAED) patterns were acquired using lacey carbon supported gold grids in a JEOL JEM-2100F electron microscope operating at 200 kV. All postelectrochemical characterizations were performed after immediate drying and storage of the samples under a vacuum N_2_ atmosphere.

### 4.4 NO_3_RR Electrochemical Measurements

The electrocatalytic performance in NO_3_RR was studied in a custom-made H-type cell (Figure S31) controlled by a Metrohm Autolab PGSTAT302N instrument, using a reversible hydrogen electrode as the reference electrode (RE), CA- and CV-reconstructed electrocatalyst as the working electrode (WE), and Pt mesh (3.0 cm × 1.0 cm × 0.1 mm) as the counter electrode (CE). The catholyte was 20 mL of 1.0 mol L^–1^ NaOH as the nitrate-free electrolyte and 1.0 mol L^–1^ NaOH + 20 mmol L^–1^ NaNO_3_ as the nitrate-containing electrolyte (previously purged with high-purity argon for at least for 15 min), and the anolyte was 15 mL of 1.0 mol L^–1^ NaOH. The anode and cathode compartments were separated by a Fumasep FAB-PK-130 membrane to suppress possible Pt redeposition on the working electrode and to avoid oxidation of NO_3_RR intermediates and products. The CVs were first acquired in NaOH, and after obtaining reproducible voltammograms (after at least three cycles), the electrochemical surface area (ECSA) was determined using the double-layer capacitance (*C*_dl_) measured by CV in a nonfaradaic region (Figure S8) and a specific capacitance (*C*_s_) of 40 μF cm^–2^ as commonly reported for oxide-based electrocatalysts.^28,42^ Then, the polarization curve was obtained by linear sweep voltammetry (LSV) from 0.40 to −0.40 V_RHE_ in a nitrate-free electrolyte at a 1 mV s^–1^ scan rate. Nitrate was then added to the alkaline electrolyte, and the nitrate-containing LSV recorded. 1 h electrolysis experiments were carried out in chronoamperometric mode from 0.0 to −0.40 V_RHE_ at every 100 mV under controlled mass transport by a rotation rate of 400 rpm. A long-term stability test was conducted at −0.30 V_RHE_ in 6 consecutive 2 h electrolysis cycles. At each electrolysis, the electrolyte was collected for product quantification and replaced with fresh electrolyte. During the electrolyte renewal, no potential was applied, and the electrode was minimally exposed to air.

### 4.5 NH_3_ and NO_2_^–^ Quantification

Ammonia and nitrite were quantified by colorimetric methods using a Bel Photonics UV-M51 spectrophotometer. NO_2_^–^ was quantified by the Griess method. 30 μL of the catholyte was diluted with 3.0 mL of water and 1.0 mL of the chromogenic solution, prepared by the dissolution of 0.1 g of *N*-(1-naphthyl)­ethylenediamine, 1.0 g of sulfanilamide, and 10 mL of phosphoric acid in 100 mL of water. The UV–vis spectra were acquired from 400 to 650 nm, and the maximum at 547 nm was used for quantification based on the analytical curve with an excellent correlation coefficient (Figure S9). Merck Spectroquant NH_4_-N (lot number HC200877) was used for NH_3_ quantification. Briefly, an aliquot from the catholyte (50–100 μL) was diluted in 10 mL of water. Then, 5 mL of the coloring agents was added, and after 5 min, the absorption spectra were recorded between 550 and 850 nm at a 1 nm step. The maximum at 690 nm was used for quantification in accordance with the analytical curve with an excellent correlation coefficient (Figure S10).

### 4.6 Faradaic Efficiencies and Yield Rate Calculation

Faradaic efficiencies (FE) were determined by the charge consumption for ammonia and nitrite production according to eq 1, and the yield rates (YR) for both species were calculated by eq 2.

1 $$\mathrm{FE}=\frac{n\times F\times c\times V}{i\times t}\times 100\%$$

2 $$\mathrm{YR}=\frac{c\times V}{t\times A}$$

where *n* is the number of electrons transferred in the half-reaction (8 for NH_3_ and 2 for NO_2_^–^), *F* is the Faraday constant (96 500 C mol^–1^), *c* is the concentration in mol L^–1^, *V* is the catholyte volume (0.020 L), *i* is the total current, *t* is the electrolysis duration, and *A* is the geometric area (cm^2^).

### 4.7 SFC-ICP-MS Measurements

The online dissolution of the metals during the electrochemical measurements was performed using a scanning flow cell (SFC) coupled to an inductively coupled plasma mass spectrometry (ICP-MS) system. The SFC was composed of three intersecting channels. One channel was dedicated to the connection of the reference electrode (RE) (Ag/AgCl 3 M KCl, Metrohm, Switzerland). The V-shaped channel connected the working electrode (WE) (from the bottom opening, *A* = 0.00907 cm^2^) with the glassy carbon counter electrode (CE) and the electrolyte outlet for the ICP-MS (NexION 350X, PerkinElmer). On the counter electrode channel, an external Y-connector was used to connect the electrolyte inlet. To the bottom opening, a silicon ring was assembled to prevent electrolyte leakage when the cell is in contact with the working electrode. The SFC was assembled to a force sensor in a fixed position over an *xyz*-translational stage (Physik Instrumente, Germany) that held the working electrode and enabled precise positioning of the sample. Before the electrochemical protocol was applied, the electrolyte was pumped into the SFC, and all compartments were filled. The flow was then adjusted to achieve a stable meniscus (around 2.9 μL s^–1^), and the electrical contact was established by moving the working electrode in the *z*-axis until the desired force was achieved (1.0–1.2 N). For each measurement, a fresh spot on the sample was selected. The experimental apparatus is depicted in Figure 7 (more details about the SFC-ICP-MS setup can be found in previously published work).^80^

**Figure 7:**
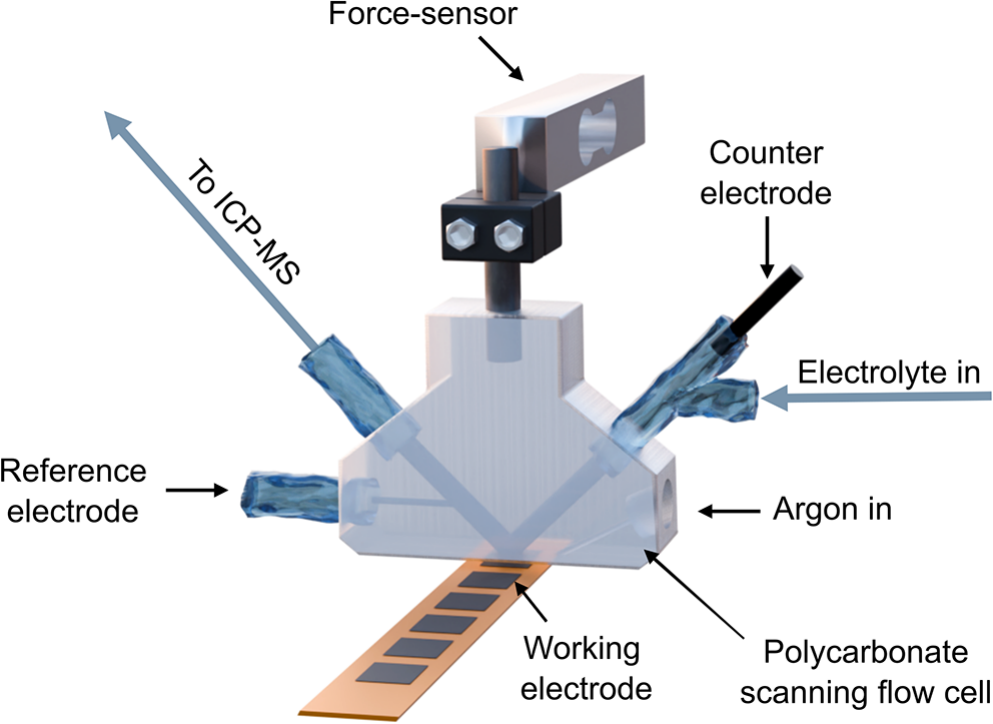
Scheme of the experimental apparatus for a scanning flow cell coupled to ICP-MS experiments.

The ICP-MS online analysis was performed by connecting the electrolyte from the outlet channel and mixing it with an internal standard (IS) prepared in a 2% HNO_3_ solution containing ^74^Ge (50 μg L^–1^), an isotopic tracer for the quantification of metals (Co and Cu). The ICP-MS was calibrated daily using a four-point calibration slope with standard solutions containing ^58^Co and ^63^Cu isotopes with a concentration range of 0–5 μg L^–1^. All measurements were carried out in an Ar-saturated atmosphere. KOH and KNO_3_ solutions were used instead of NaOH and NaNO_3_ to avoid interference with ^40^Ar^+^Na.

To understand Co and Cu dissolution during electrochemical surface activation, two protocols were used in 0.02 mol L^–1^ KOH: (i) 5 min at *j*_geo_ = 0.00 mA cm^–2^ (∼0.80 V_RHE_) followed by 10 scan cycles of CV from 0.15 to −0.40 V_RHE_ at a 20 mV s^–1^ scan rate and ending with 5 min at *j*_geo_ = 0.00 mA cm^–2^; and (ii) 300 s at *j*_geo_ = 0.00 mA cm^–2^ followed by 25 min at −0.30 V_RHE_. The influence of nitrate was assessed using the following third procedure: 5 min at *j*_geo_ = 0.00 mA cm^–2^ and 1 scan cycle of CV from 0.85 to −0.40 V_RHE_ at 2 mV s^–1^ in 0.02 mol L^–1^ KOH and 0.02 mol L^–1^ KOH + 0.02 mol L^–1^ KNO_3_.

### 4.8 Thin Layer *In Situ* FTIR

Attenuated total reflectance (ATR) configuration in a thin electrolyte layer was used to acquire the spectra. To this end, a three-electrode cell (reference electrode (RE): reversible hydrogen electrode; counter electrode (CE): Pt foil (1.0 cm × 1.0 cm × 0.1 mm); and working electrode (WE): CA-reconstructed electrocatalyst) was mounted and positioned under a moving mirror accessory (Specac Gateway ATR accessory) in a ThermoNicolet Nexus 670 spectrometer by using a Si ATR trapezoidal crystal. Spectra were collected from 4000 to 1200 cm^–1^, with a 8 cm^–1^ resolution and 32 accumulations according to the spectra at open circuit potential as the reference spectrum in 0.01 mol L^–1^ NaOH and 0.05 mol L^–1^ NaNO_3_.

### 4.9 Online DEMS

Online differential electrochemical mass spectrometry experiments were conducted by coupling chronoamperometry measurements to the mass spectrometer to track possible gaseous and volatile species by their ionic currents of mass/charge (*m*/*z*) of 2 (H_2_^+^), 17 (NH_3_^+^), 28 (N_2_^+^), 29 (N_2_H^+^), 30 (NO^+^), 32 (N_2_H_4_^+^), 33 (NH_2_OH^+^), and 44 (N_2_O^+^). The working electrode (WE) was a CA-reconstructed catalyst prepared on Cu mesh (Alfa Aesar, copper gauze, 50 mesh woven from a 0.23 mm diameter wire) and PTFE membrane (Gore-Tex, 0.02 μm pore size and 50 μm thickness) underneath, attached to a PTFE holder and screwed to the stainless-steel flange, which was placed inside a custom-made electrochemical cell. A platinum mesh and a reversible hydrogen electrode were used as counter (CE) and reference electrodes (RE), respectively. The experiments were conducted in Ar-saturated 1.0 mol L^–1^ NaOH and 0.1 mol L^–1^ NaNO_3_. All reported ionic current values are relative and refer to the deviation from the baseline observed during the applied potential. The acquisition frequency of different *m*/*z* values was 100 ms.

### 4.10 *In Situ* Co and Cu K-Edge XANES

Synchrotron experiments were carried out at the Carnaúba beamline (Tarumã station) of the Sirius facility using a nanofocused beam of approximately 200 × 500 nm^2^ and an estimated flux of ∼10^9^ photons s^–1^ at the sample position. X-ray fluorescence (XRF) mapping was performed in continuous scan mode (called flyscan) over a 20 × 20 μm^2^ area with a step of 400 nm (pixel size), achieved by scanning the sample relative to the fixed beam. *In situ* localized XANES and spatially resolved XANES experiments were performed in fluorescence mode using a four-bounce Si(111) monochromator (Δ*E*/*E* ≈ 10^–4^) with an energy step of 0.5 eV. All spectra were collected during chronoamperometry measurements using an EC301 potentiostat (Stanford Research Systems) in an electrolyte containing 1.0 mol L^–1^ NaOH and 20 mmol L^–1^ NaNO_3_, initially at OCP and then under applied potentials (i) from 0.00 to −0.40 V_RHE_ in 100 mV increments for localized XANES and (ii) at −0.20 V_RHE_ for spatially resolved acquisition. In each case, the steady-state current was allowed to stabilize prior to the spectra collection.

## Supplementary Material



## Notes

Additional experimental details; synthesis schematics; SEM, TEM, HAADF-STEM, SAED, and EDX microstructural analyses; XRD patterns; XPS survey spectra and surface composition; electrochemical measurements including double-layer capacitance determination, NO_3_RR performance at varying nitrate concentrations, and control experiments for hydrazine reduction; comparison of catalytic performance with previously reported catalysts; NH_3_ and NO_2_^–^ calibration curves; *in situ* XRF maps; Co and Cu K-edge XANES spectra; and Pourbaix diagrams (PDF)
