# Supplementary material for: Tailoring Reconstruction of Co/Cu Mixed Oxide-Derived Tandem Electrocatalysts via In Situ Electrochemical Dissolution–Redeposition for Enhanced Nitrate-to-Ammonia Conversion
Source: JACS Au. 2026 Jan 9;6(2):1020–35. doi: 10.1021/jacsau.5c01439 (PMC12933307; doi:10.1021/jacsau.5c01439)
Supplement: Supplementary file 1 [file au5c01439_si_001.pdf]

Supporting Information

Tailoring reconstruction of Co/Cu mixed oxide-derived tandem electrocatalysts via *in situ* electrochemical dissolution–redeposition for enhanced nitrate-to-ammonia conversion

Manuel E. G. Winkler,*[a,b,c] Rafael G. Yoshimura,[a,d] Pâmella S. Rodrigues,[c,e] Matheus P. Sales,[a] Kauan L. Gomes,[a] Itamar T. Neckel,[d] Santiago J. A. Figueroa,[d] João B. Souza Jr,[a,f] Edson A. Ticianelli,[e] Nirala Singh,[g] Fabio H. B. Lima,[e] Serhiy Cherevko,[c] Raphael Nagao*[a,b]

[a]*Institute of Chemistry, University of Campinas, Campinas, SP, 13083-862, Brazil*
[b]*Center for Innovation on New Energies, University of Campinas, 13083-084, Campinas, SP, Brazil*
[c]*Forschungszentrum Jülich GmbH, Helmholtz-Institute Erlangen-Nürnberg for Renewable Energy (IET-2), Cauerstr. 1, 91058 Erlangen, Germany*
[d]*Brazilian Synchrotron Light Laboratory, Brazilian Center for Research in Energy and Materials, Campinas, SP, 13083-100, Brazil*
[e]*São Carlos Institute of Chemistry, University of São Paulo, São Carlos, SP, 13560-970, Brazil*
[f]*Brazilian Nanotechnology National Laboratory, Brazilian Center for Research in Energy and Materials, Campinas, SP, 13083-100, Brazil*
[g]*Department of Chemical Engineering, University of Michigan, Ann Arbor, Michigan 48109-2136, USA*

Corresponding authors: manuelgw@unicamp.br and nagao@unicamp.br

LIST OF FIGURES

Figure S1. Schematic illustration of the synthesis of the Co_3O_4/Cu_xO tandem electrocatalyst via Co electrodeposition, CoC_2O_4 growth, and calcination in air. 5

Figure S2. SEM image of electrodeposited Co at (a) low and (b) high magnification. ...5

Figure S3. SEM image of electrochemically grown CoC_2O_4...5

Figure S4. (a) SEM and corresponding (b) SEM-EDX elemental maps of Co (purple) and Cu (yellow) of the Cu-rich region of Co_3O_4/Cu_xO pre-catalyst. 5

Figure S5. (a) SEM and (b) corresponding Co and Cu SEM-EDX elemental maps of Co_3O_4/Cu_xO tandem electrocatalyst. Scale bars correspond to 20 μm. (c) EDX spectra obtained from Figure S1b...6

Figure S6. (a) Edge-positioned SEM micrograph and corresponding (b) Co and Cu SEM-EDX elemental maps of the Co_3O_4/Cu_xO edge. Scale bars correspond to 20 μm.6

Figure S7. Survey XPS spectra of Co_3O_4/Cu_xO and surface chemical composition. 7

Figure S8. (a) Cyclic voltammograms (CVs) for the determination of the double-layer capacitance and (b) plot of the current densities *versus* scan rate. 7

Figure S9. (a) UV-vis spectra of NO_2^- quantification (from 0.0 ppm to 1.2 ppm) and (b) corresponding analytical curve. 8

Figure S10. (a) UV-vis spectra of NH_4-N quantification (from 0.0 ppm to 2.0 ppm) and (b) corresponding analytical curve. 8

Figure S11. Cyclic voltammograms (CVs) for the determination of the double-layer capacitance and plots of the current densities *versus* scan rate of (a-b) CV-reconstructed and (c-d) CA-reconstructed Co/Cu mixed oxide-derived electrocatalysts..9

Figure S12. NO_3RR performance of CA- and CV-reconstructed Co/Cu mixed oxide-derived electrocatalyst at different nitrate concentrations in 1.0 mol L^{-1} NaOH at -0.20 V_{RHE}. (a) FE to NH_3 and NO_2^- and (b) NH_3 yield rate..............................9

Figure S13. 1-h chronoamperometric curve of CA-reconstructed electrocatalyst at -0.30 V_{RHE} in nitrate-free (1 mol L^{-1} NaOH) and nitrate-containing electrolyte (1 mol L^{-1} NaOH + 20 mmol L^{-1} $NaNO_3$). 11

Figure S14. Cu LMM spectra of Co_3O_4/Cu_xO pre-catalyst, CA- and CV-reconstructed Co/Cu mixed oxide-derived electrocatalyst. 11

Figure S15. EDX spectra of CA- and CV-reconstructed Co/Cu mixed oxide-derived electrocatalyst from Figure 3b,d...12

Figure S16. (a) SEM image of the CA-reconstructed Co/Cu mixed oxide-derived electrocatalysts, (b) overlayed SEM/EDX elemental distribution of Co and Cu, and (c-d) the individual SEM/EDX Co and Cu elemental maps, respectively....12

Figure S17. (a) SEM image of the CV-reconstructed Co/Cu mixed oxide-derived electrocatalysts, (b) overlayed SEM/EDX elemental distribution of Co and Cu, and (c-d) the individual SEM/EDX Co and Cu elemental maps, respectively....13

Figure S18. (a) XPS survey spectra of CA- and CV-reconstructed Co/Cu mixed oxide-derived electrocatalyst and (b) the respective surface composition. 13

Figure S19. XRD pattern of CA- and CV-reconstructed Co/Cu mixed oxide-derived electrocatalysts..14

Figure S20. (a) TEM image, (b) HAADF-STEM and corresponding EDX elemental maps of (c) Co, (d) O, and (e) Cu of a CA-reconstructed Co/Cu mixed oxide-derived nanowire. 15

Figure S21. (a) TEM image, (b) HAADF-STEM and corresponding EDX elemental maps of (c) Co, (d) O, and (e) Cu of a CV-reconstructed Co/Cu mixed oxide-derived nanowire. 15

Figure S22. *In situ* XRF maps of (a) Co an (b) Cu of Co_3O_4-Cu_xO at OCP in 1.0 mol L^{-1} NaOH and 20 mmol L^{-1} $NaNO_3$..17

Figure S23. *In situ* single-point (a) Co K-edge spectra and (b) 1st Derivative of Co/Cu mixed oxide-derived electrocatalyst in 1.0 mol L^{-1} NaOH + 20 mmol L^{-1} $NaNO_3$ and cobalt-based standard samples...17

Figure S24. *In situ* Cu K-edge spectra of Co/Cu mixed oxide-derived electrocatalyst in 1.0 mol L^{-1} NaOH + 20 mmol L^{-1} $NaNO_3$ and copper oxides standard samples. 18

Figure S25. Pourbaix diagram of Co constructed using experimental thermodynamic data[10] at 25 °C, assuming an aqueous ion concentration of 10^{-9} mol L^{-1}. The potential range used in this study corresponds to 0.073 V_{SHE} to −1.127 V_{SHE} at pH 12.3. The dashed black lines outline the stability regions of the Co species based on the calculated predominance of dissolved forms:...19

Figure S26. Pourbaix diagram of Cu constructed using experimental thermodynamic data[10] at 25 °C, assuming an aqueous ion concentration of 10^{-9} mol L^{-1}. The potential range used in this study corresponds to 0.073 V_{SHE} to −1.127 V_{SHE} at pH

12.3. The dashed gray lines outline the stability regions of the Cu species based on the calculated predominance of dissolved forms:..20

Figure S27. (a) Cyclic voltammogram of CA-reconstructed Co/Cu mixed oxide-derived electrocatalyst in 1.0 mol L^{-1} NaOH and 0.20 mol L^{-1} N_2H_4 from 0.15 V_{RHE} to −0.40 V_{RHE} at 1 mV s^{-1} scan rate; (b) chronoamperometric curve of hydrazine reduction at −0.40 V_{RHE}; and (c) post-electrolysis spectrophotometric detection of ammonia. Inset: photograph of the tested electrolyte..21

Figure S28. SEM/EDX Co and Cu elemental maps of (a) pristine and (b) after 1-CV in 0.02 mol L^{-1} NaOH from 0.80 V_{RHE} to −0.40 V_{RHE} at 2 mV s^{-1} scan rate. 21

Figure S29. SEM/EDX Co and Cu elemental maps of (a) pristine and (b) after 1-CV in 0.02 mol L^{-1} NaOH + 0.02 mol L^{-1} $NaNO_3$ from 0.80 V_{RHE} to −0.40 V_{RHE} at 2 mV s^{-1} scan rate..21

Figure S30. (a-b) SEM image of Co/Cu mixed-oxide electrocatalyst after 1-CV cycle in nitrate-containing electrolyte (0.02 mol L^{-1} NaOH + 0.02 mol L^{-1} $NaNO_3$) from 0.80 V_{RHE} to −0.40 V_{RHE} at 2 mV s^{-1} scan rate. 22

Figure S31. Custom-made H-type electrochemical used in the NO_3RR experiments. RE: reversible hydrogen electrode; WE: CA- and CV-reconstructed electrocatalyst; and CE: Pt mesh. 22

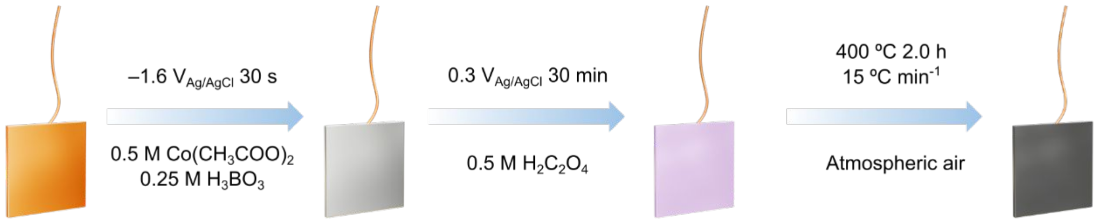

Figure S1. Schematic illustration of the synthesis of the Co_3O_4/Cu_xO tandem electrocatalyst via Co electrodeposition, CoC_2O_4 growth, and calcination in air.

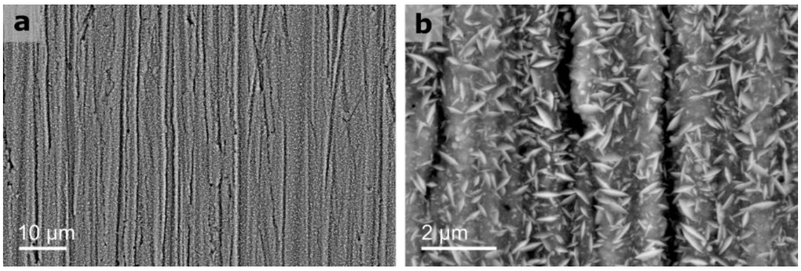

Figure S2. SEM image of electrodeposited Co at (a) low and (b) high magnification.

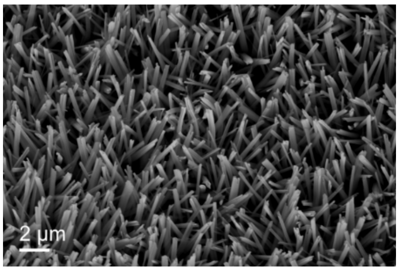

Figure S3. SEM image of electrochemically grown CoC_2O_4.

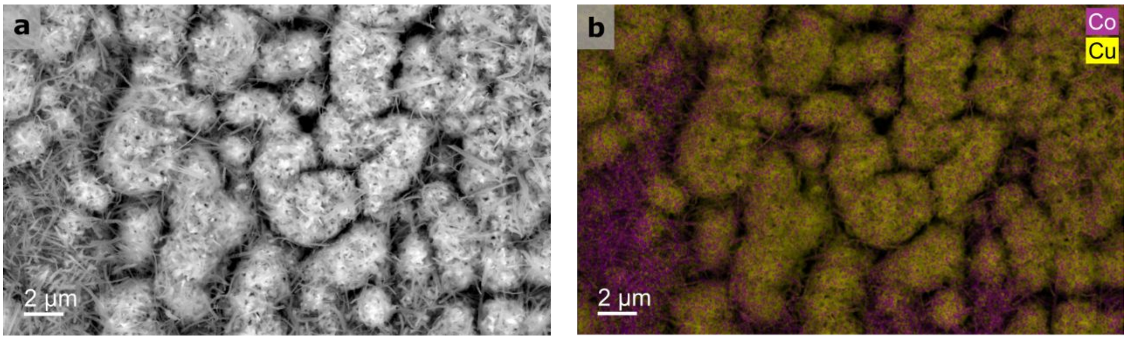

Figure S4. (a) SEM and corresponding (b) SEM-EDX elemental maps of Co (purple) and Cu (yellow) of the Cu-rich region of Co_3O_4/Cu_xO pre-catalyst.

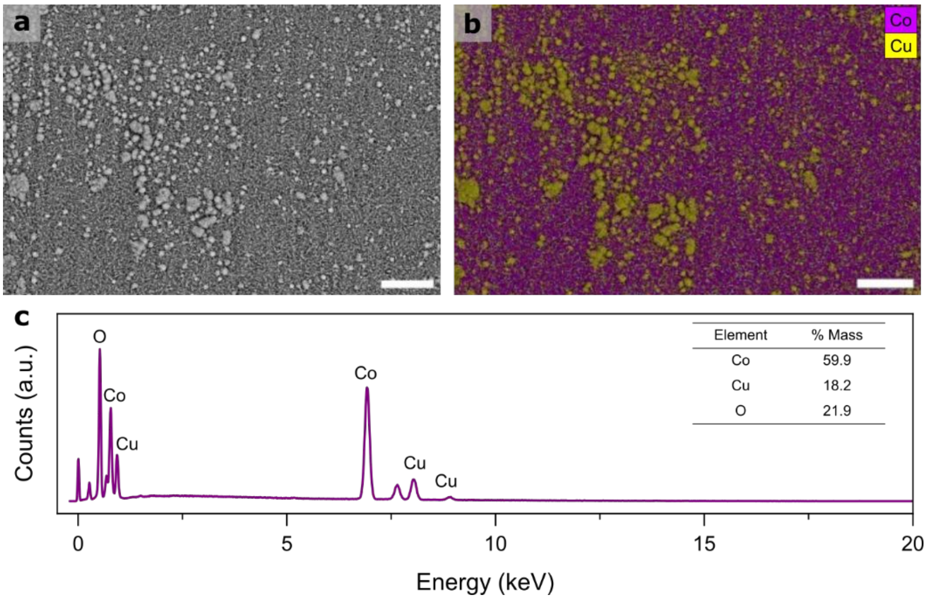

Figure S5. (a) SEM and (b) corresponding Co and Cu SEM-EDX elemental maps of Co_3O_4/Cu_xO tandem electrocatalyst. Scale bars correspond to 20 µm. (c) EDX spectra obtained from Figure S1b.

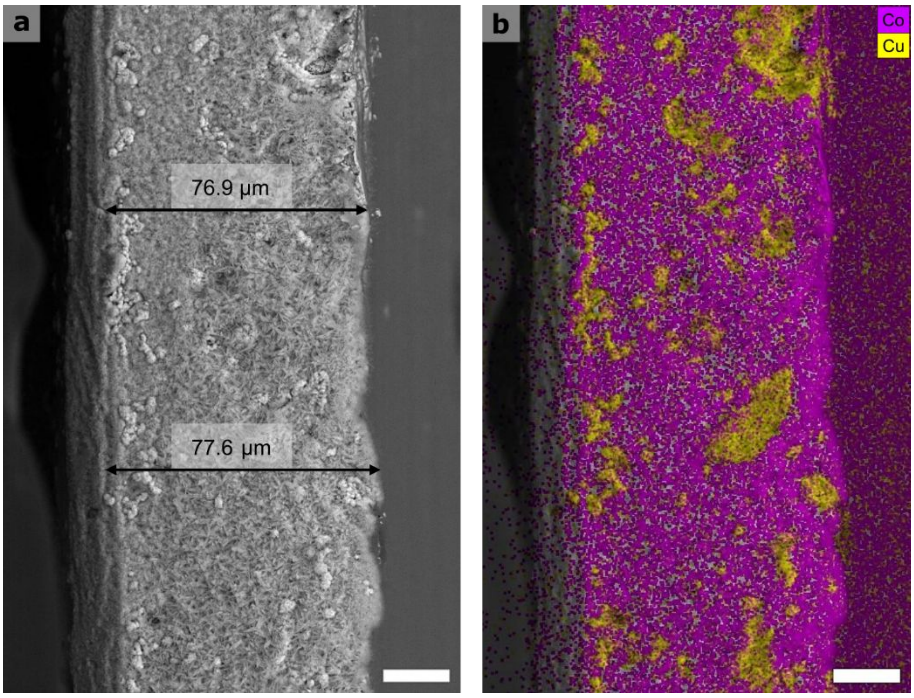

Figure S6. (a) Edge-positioned SEM micrograph and corresponding (b) Co and Cu SEM-EDX elemental maps of the Co_3O_4/Cu_xO edge. Scale bars correspond to 20 µm.

Table S1. d-spacing and plane assignment from the SAED pattern of pre-catalyst Co_3O_4/Cu_xO.

d-spacing (Å)	Assignment	Plane
4.62	Co_3O_4[a]	(111)[a]
2.87	Co_3O_4[a], Cu_2O[b]	(220)[a], (110)[b]
2.44	Co_3O_4[a], Cu_2Ob	(311)[a], (111)[b]
2.04	Co_3O_4[a], Cu[b]	(400)[a], (111)[c]
1.57	Co_3O_4[a], Cu_2O[b]	(511)[a], (220)[b]
1.40	Co_3O_4[a]	(440)[a]

[a]Co_3O_4 JCPDS: 42-1467;
[b]Cu_2O JCPDS: 5-0667;
[c]Cu JCPDS: 4-0836.

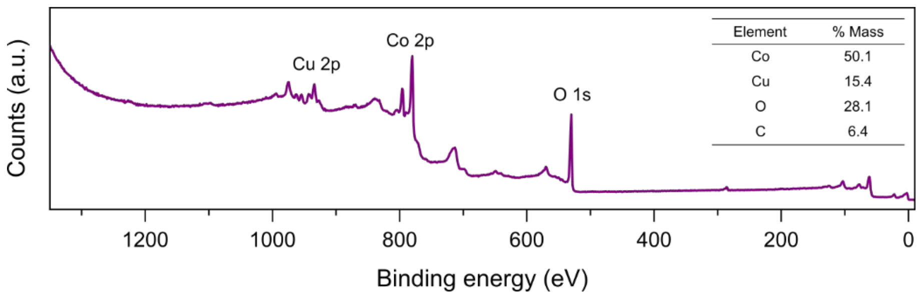

Figure S7. Survey XPS spectra of Co_3O_4/Cu_xO and surface chemical composition.

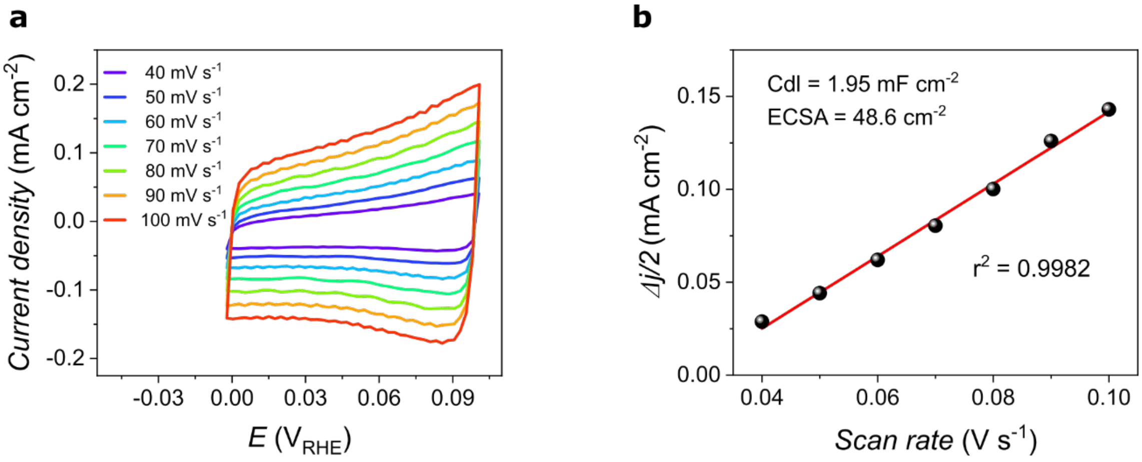

Figure S8. (a) Cyclic voltammograms (CVs) for the determination of the double-layer capacitance and (b) plot of the current densities *versus* scan rate.

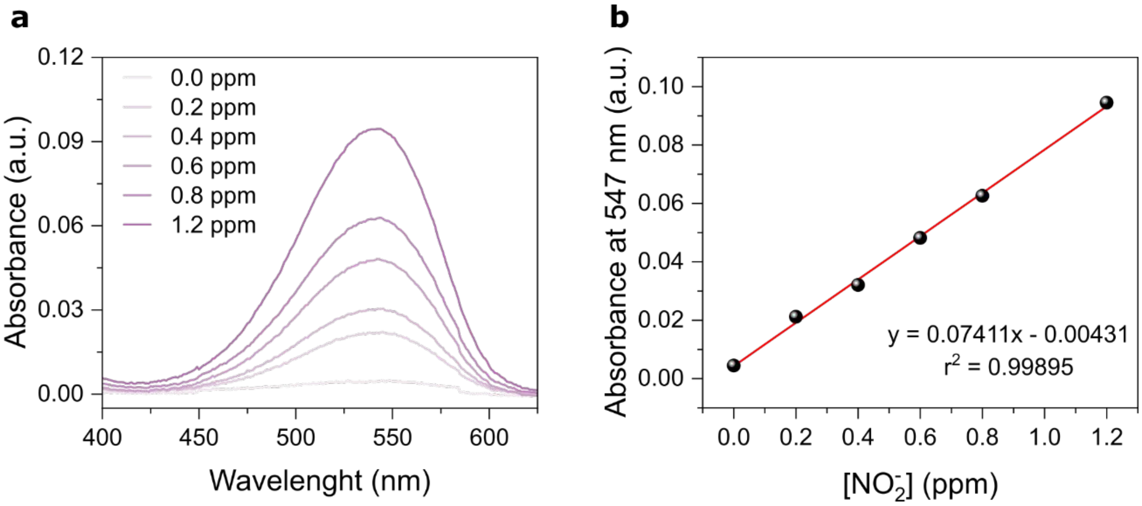

Figure S9. (a) UV-vis spectra of NO_2^- quantification (from 0.0 ppm to 1.2 ppm) and (b) corresponding analytical curve.

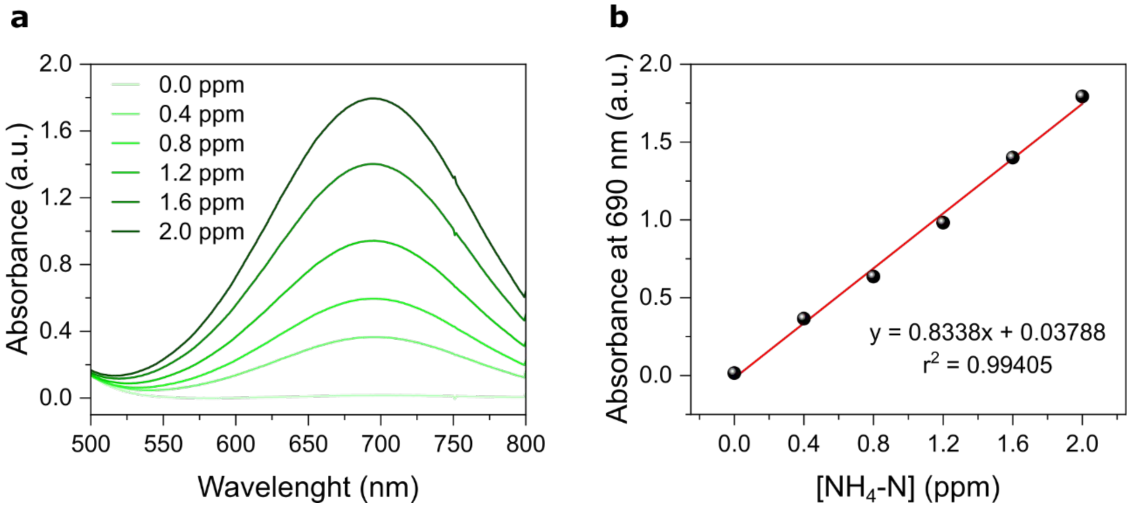

Figure S10. (a) UV-vis spectra of NH_4-N quantification (from 0.0 ppm to 2.0 ppm) and (b) corresponding analytical curve.

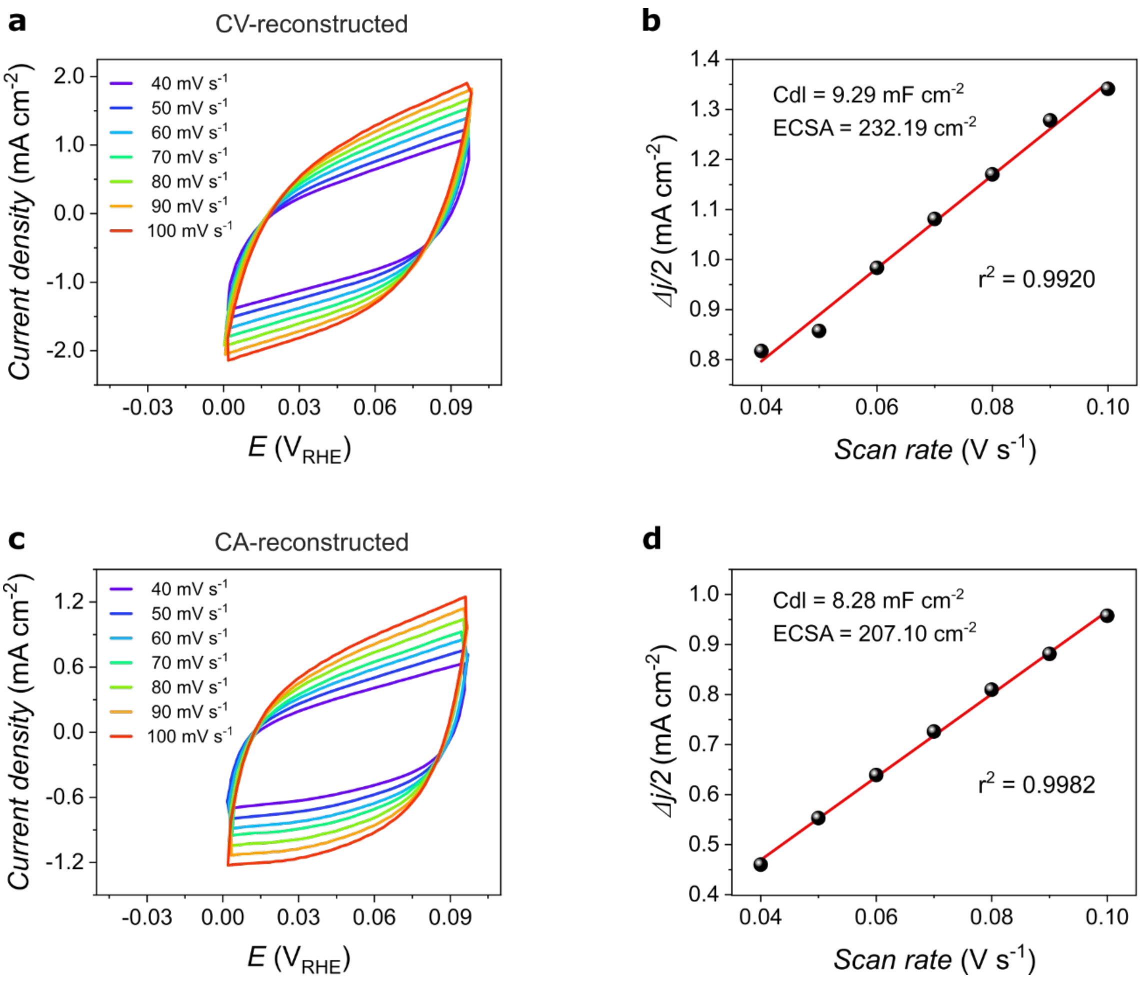

Figure S11. Cyclic voltammograms (CVs) for the determination of the double-layer capacitance and plots of the current densities *versus* scan rate of (a-b) CV-reconstructed and (c-d) CA-reconstructed Co/Cu mixed oxide-derived electrocatalysts.

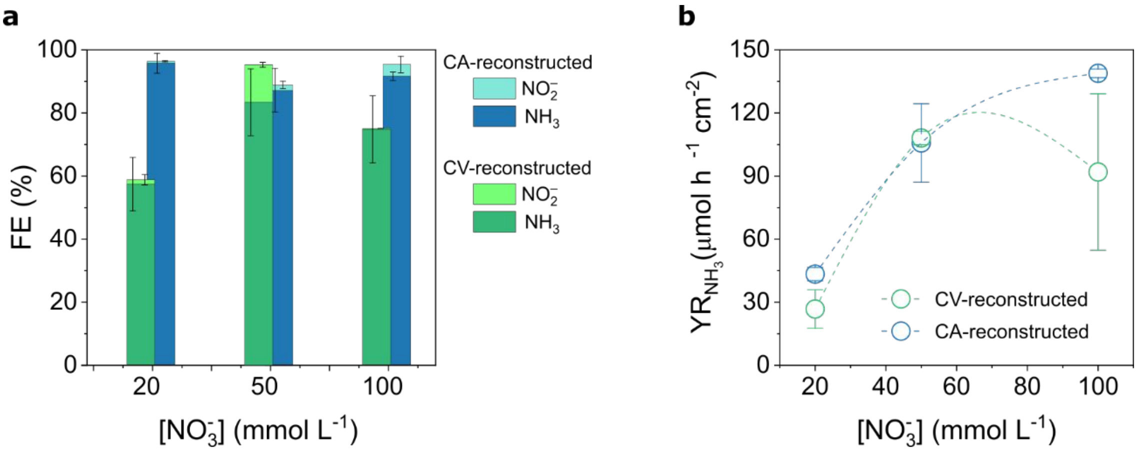

Figure S12. NO_3RR performance of CA- and CV-reconstructed Co/Cu mixed oxide-derived electrocatalyst at different nitrate concentrations in 1.0 mol L^{-1} NaOH at –0.20 V_{RHE}. (a) FE to NH_3 and NO_2^- and (b) NH_3 yield rate.

Table S2. Comparison of catalytic performance of CA- and CV-reconstructed Co/Cu mixed oxide-derived electrocatalyst with others reported Co and Cu oxide-derived catalyst for nitrate electroreduction to ammonia.

Catalyst	Electrolyte	Performance	E_{RHE} (V)	Ref.
CV-reconstructed Co/Cu mixed oxide-derived	1 mol L^{-1} NaOH + 0.1 mmol L^{-1} $NaNO_3$	FE = 80.4 ± 10.6 % YR = 108.1 ± 3.2 μmol h^{-1} cm^{-2}	−0.2	This work
CA-reconstructed Co/Cu mixed oxide-derived	1 mol L^{-1} NaOH + 0.1 mmol L^{-1} $NaNO_3$	FE = 91.6 ± 1.3 % YR = 139 ± 2.0 μmol h^{-1} cm^{-2}	−0.2	This work
Cu_2O (111)	1 mol L^{-1} NaOH + 0.1 mol L^{-1} $NaNO_3$	FE = 76 %	−0.3	[1]
Cu_2O (100)	1 mol L^{-1} NaOH + 0.1 mol L^{-1} $NaNO_3$	FE = 68 %	−0.3	[1]
Cu_2O nanocubes	1 mol L^{-1} NaOH + 14 mmol L^{-1} $NaNO_3$	FE = 94 % YR = 0.149 mmol h^{-1} cm^{-2}	−0.3	[2]
Cu_2O nanocubes	0.1 mol L^{-1} Na_2SO_4 + 8 mmol L^{-1} $NaNO_3$	FE = 88 % YR = 45 μmol h^{-1} cm^{-2}	−0.3	[3]
Co_3O_4 (111) nanosheets	0.1 mol L^{-1} K_2SO_4 + 500 ppm KNO_3	FE = 99 % YR = 5.73 mg mg_{cat}^{-1} h^{-1}	−0.7 −0.9	[4]
Core-shell Cu/CuO_x and Co/CoO	0.1 mol L^{-1} KOH 0.1 mol L^{-1} KNO_3	FE = 93.3 % YR = 1.17 mmol h^{-1} cm^{-2}	−0.175	[5]
CoCuP	0.1 M K_2SO_4 + 10 mM KNO_3	FE = 89.2 YR = 2.91 mg h^{-1} cm^{-2}	−0.5	[6]
Cu/Cu_2O/Co_3O_4	0.5 mol L^{-1} Na_2SO_4 + 10 mmol L^{-1} $NaNO_3$	FE = 91.5 YE = 5.7 mg h^{-1} mg_{cat}^{-1}	−0.6	[7]
Cu-Co/BPC	0.1 mol L^{-1} Na_2SO_4 + 0.1 mol L^{-1} $NaNO_3$	FE = 84.5 ± 1.6% YR = 6 mg h^{-1} cm^{-2}	−1.0	[8]
Cu_2O@CoO yolk-shell	1.0 mol L^{-1} KOH 0.1 mol L^{-1} KNO_3	FE = 98.7 % YR = 0.192 mg h^{-1} cm^{-2}	−0.2	[9]

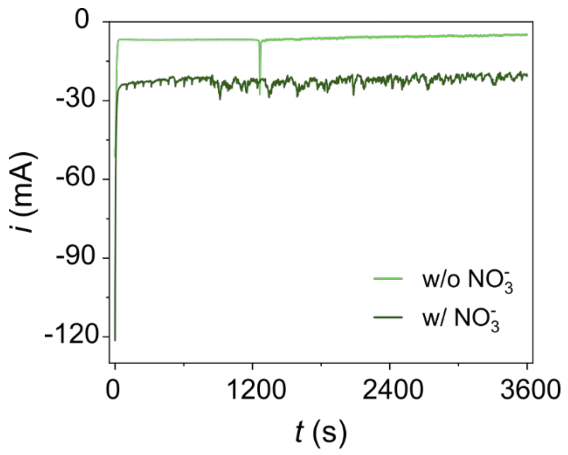

Figure S13. 1-h chronoamperometric curve of CA-reconstructed electrocatalyst at –0.30 V_{RHE} in nitrate-free (1 mol L^{-1} NaOH) and nitrate-containing electrolyte (1 mol L^{-1} NaOH + 20 mmol L^{-1} $NaNO_3$).

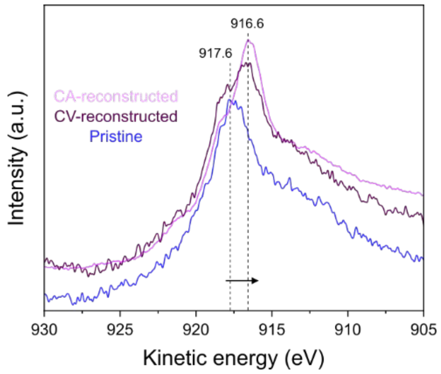

Figure S14. Cu LMM spectra of Co_3O_4/Cu_xO pre-catalyst, CA- and CV-reconstructed Co/Cu mixed oxide-derived electrocatalyst.

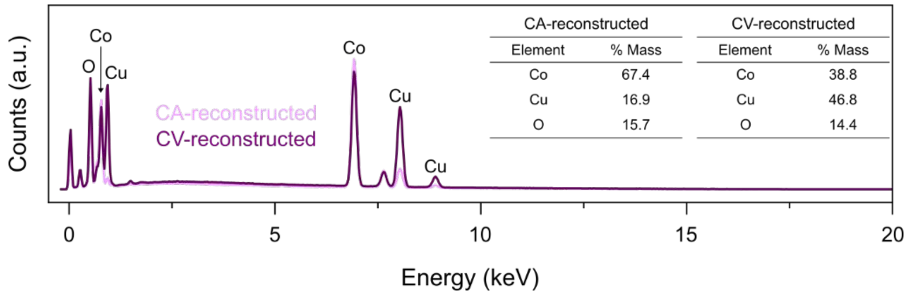

Figure S15. EDX spectra of CA- and CV-reconstructed Co/Cu mixed oxide-derived electrocatalyst from Figure 3b,d.

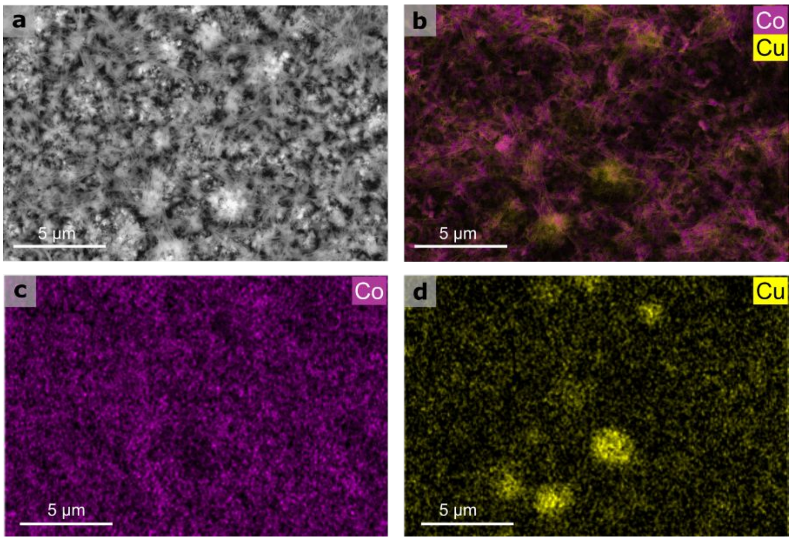

Figure S16. (a) SEM image of the CA-reconstructed Co/Cu mixed oxide-derived electrocatalysts, (b) overlayed SEM/EDX elemental distribution of Co and Cu, and (c-d) the individual SEM/EDX Co and Cu elemental maps, respectively.

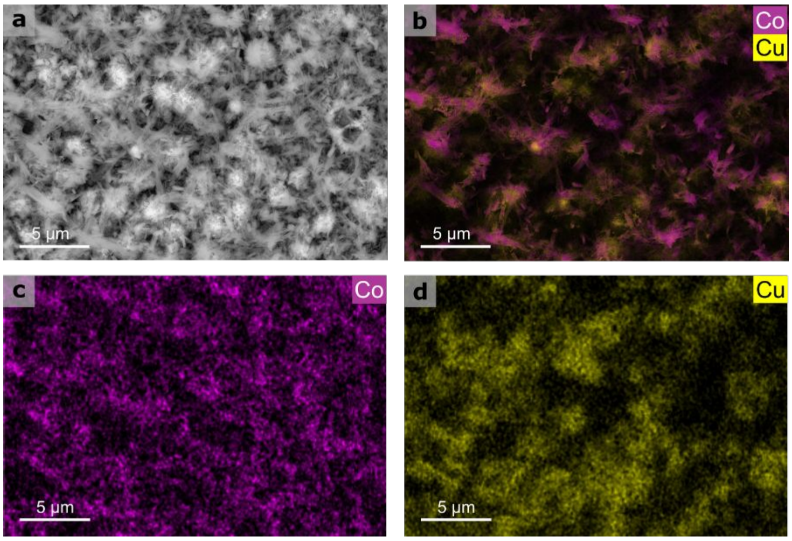

Figure S17. (a) SEM image of the CV-reconstructed Co/Cu mixed oxide-derived electrocatalysts, (b) overlayed SEM/EDX elemental distribution of Co and Cu, and (c-d) the individual SEM/EDX Co and Cu elemental maps, respectively.

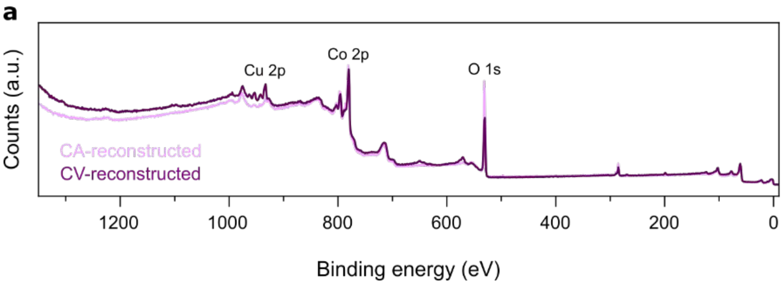

b

CA-reconstructed		CV-reconstructed	
Element	% Mass	Element	% Mass
Co	53.9	Co	47.8
Cu	3.6	Cu	13.6
O	33.0	O	30.1
C	9.5	C	8.6

Figure S18. (a) XPS survey spectra of CA- and CV-reconstructed Co/Cu mixed oxide-derived electrocatalyst and (b) the respective surface composition.

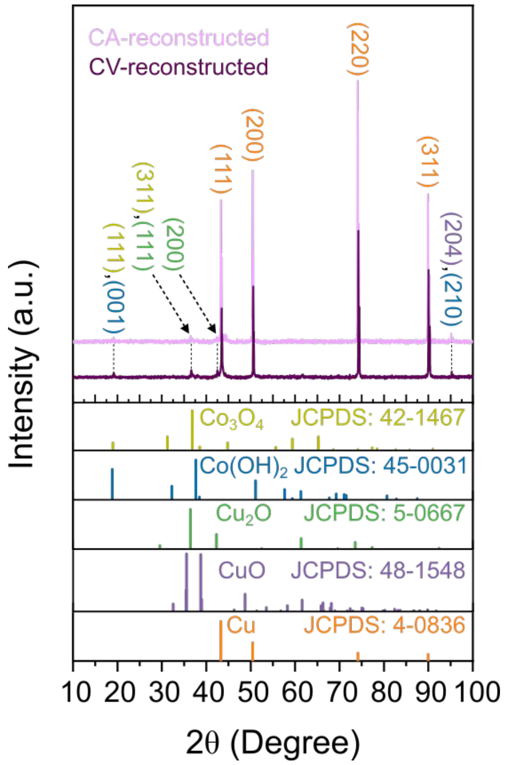

Figure S19. XRD pattern of CA- and CV-reconstructed Co/Cu mixed oxide-derived electrocatalysts.

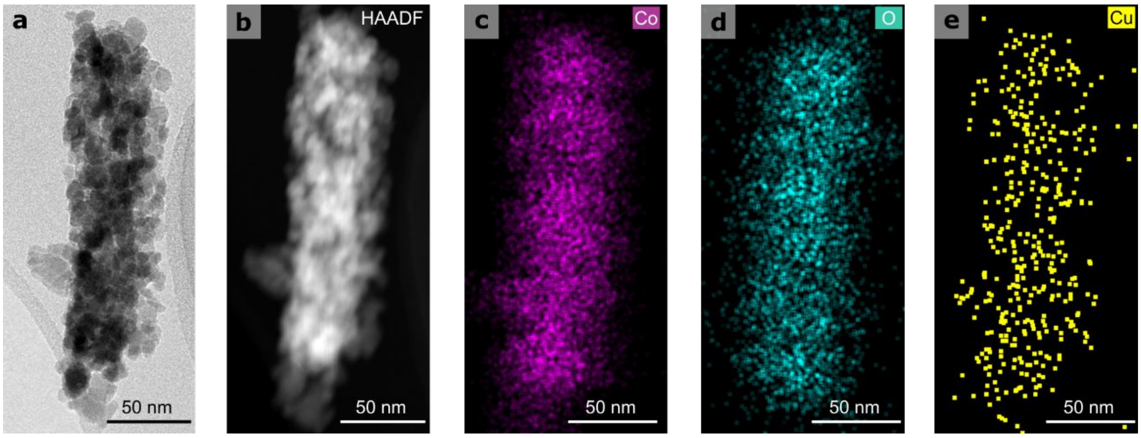

Figure S20. (a) TEM image, (b) HAADF-STEM and corresponding EDX elemental maps of (c) Co, (d) O, and (e) Cu of a CA-reconstructed Co/Cu mixed oxide-derived nanowire.

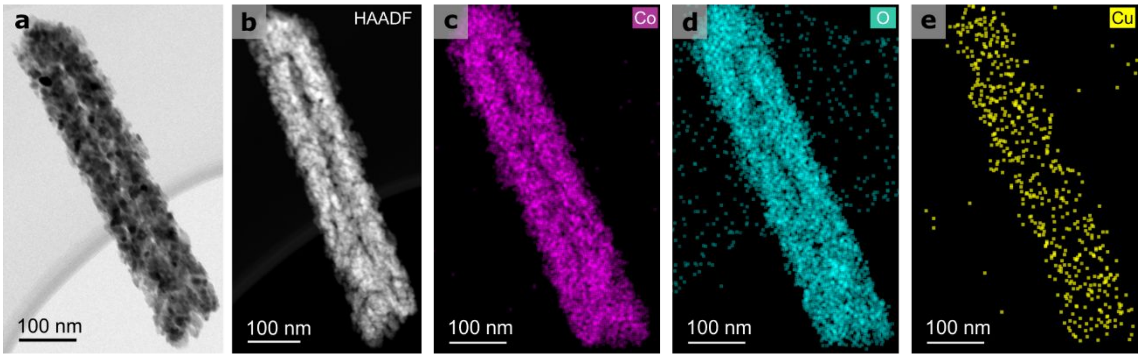

Figure S21. (a) TEM image, (b) HAADF-STEM and corresponding EDX elemental maps of (c) Co, (d) O, and (e) Cu of a CV-reconstructed Co/Cu mixed oxide-derived nanowire.

Table S3. d-spacing and plane assignment from the SAED pattern of a CA-reconstructed Co/Cu mixed oxide-derived nanowire.

d-spacing (Å)	Assignment	Plane
4.71	$Co(OH)_2^a$, $Co_3O_4^b$	$(001)^a$, $(111)^b$
2.98	$Co_3O_4^b$, Cu_2O^c,	$(220)^b$, $(110)^c$
2.53	$Co_3O_4^b$, Cu_2O^c	$(311)^b$, $(111)^c$
2.09	$Co_3O_4^b$, Cu^d	$(400)^b$, $(111)^d$
1.60	$Co(OH)_2$, Co_3O_4	$(110)^a$, $(511)^b$
1.50	$Co(OH)_2^a$, $Co_3O_4^b$, Cu_2O^c,	$(111)^a$, $(440)^b$, $(220)^c$

[a]$Co(OH)_2$ JCPDS: 45-0031
[b]Co_3O_4 JCPDS: 42-1467;
[c]Cu_2O JCPDS: 5-0667;
[d]Cu JCPDS: 4-0836.

Table S4. d-spacing and plane assignment from the SAED pattern of a CV-reconstructed Co/Cu mixed oxide-derived nanowire.

d-spacing (Å)	Assignment	Plane
4.75	$Co(OH)_2^a$, $Co_3O_4^b$	$(001)^a$, $(111)^b$
2.89	$Co_3O_4^b$, Cu_2O^c,	$(220)^b$, $(110)^c$
2.48	$Co_3O_4^b$, Cu_2O^c	$(311)^b$, $(111)^c$
2.08	$Co_3O_4^b$, Cu^d	$(400)^b$, $(111)^d$
1.60	$Co(OH)_2^a$, $Co_3O_4^b$	$(110)^a$, $(511)^b$
1.50	$Co(OH)_2^a$, $Co_3O_4^b$, Cu_2O^c,	$(111)^a$, $(440)^b$, $(220)^c$

[a]$Co(OH)_2$ JCPDS: 45-0031
[b]Co_3O_4 JCPDS: 42-1467;
[c]Cu_2O JCPDS: 5-0667;
[d]Cu JCPDS: 4-0836.

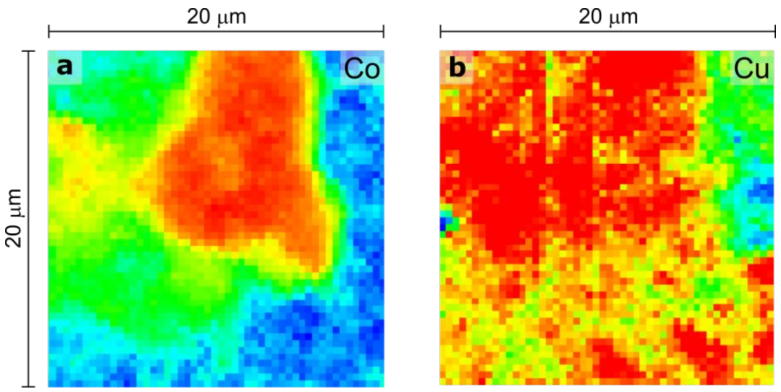

Figure S22. *In situ* XRF maps of (a) Co an (b) Cu of Co_3O_4-Cu_xO at OCP in 1.0 mol L^{-1} NaOH and 20 mmol L^{-1} $NaNO_3$.

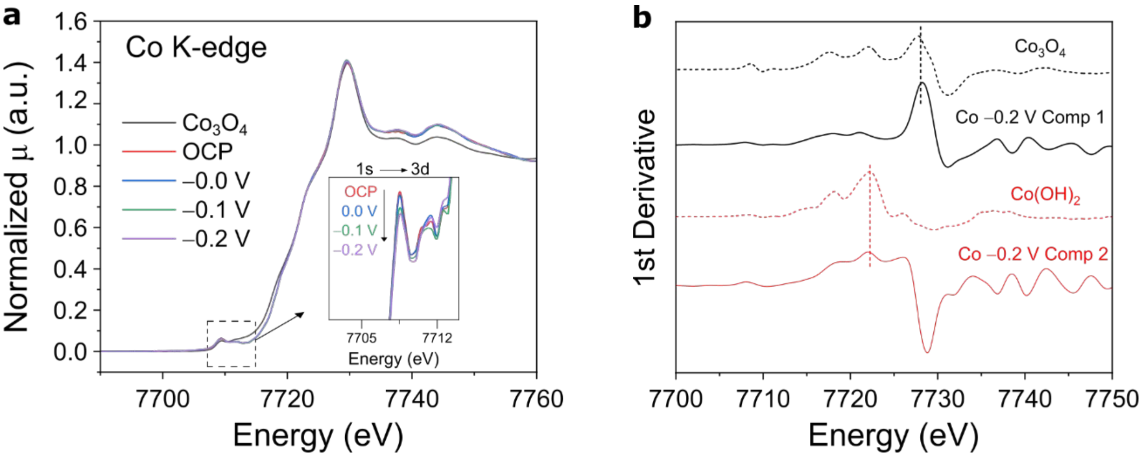

Figure S23. *In situ* single-point (a) Co K-edge spectra and (b) 1st Derivative of Co/Cu mixed oxide-derived electrocatalyst in 1.0 mol L^{-1} NaOH + 20 mmol L^{-1} $NaNO_3$ and cobalt-based standard samples.

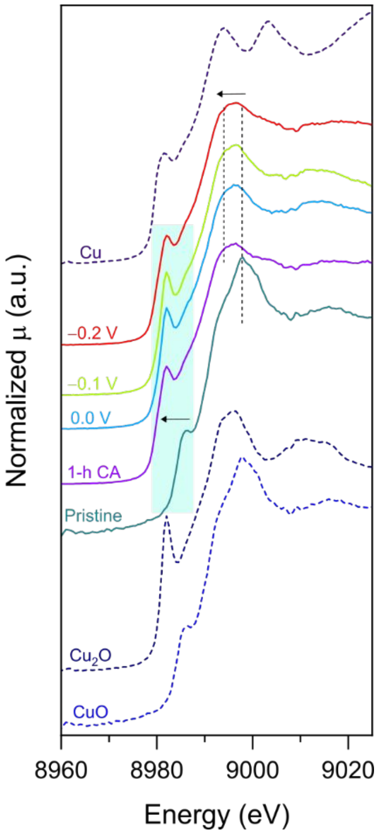

Figure S24. *In situ* Cu K-edge spectra of Co/Cu mixed oxide-derived electrocatalyst in 1.0 mol L^{-1} NaOH + 20 mmol L^{-1} $NaNO_3$ and copper oxides standard samples.

Table S5. Results from linear combination of the *in situ* Cu K-edge XANES spectra.

	Species	OCP	0.00 V_{RHE}	−0.10 V_{RHE}	−0.20 V_{RHE}
Linear combination analysis weight	Cu (0)	0.00	0.24	0.25	0.44
	Cu (I)	0.00	0.65	0.60	0.41
	Cu (II)	1.00	0.11	0.15	0.15

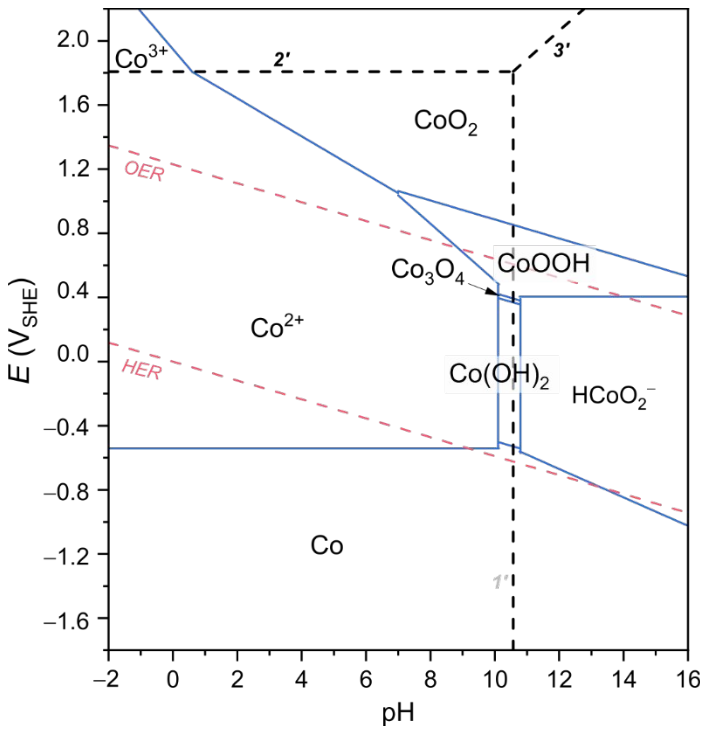

Figure S25. Pourbaix diagram of Co constructed using experimental thermodynamic data[10] at 25 °C, assuming an aqueous ion concentration of 10^{-9} mol L^{-1}. The potential range used in this study corresponds to 0.073 V_{SHE} to −1.127 V_{SHE} at pH 12.3. The dashed black lines outline the stability regions of the Co species based on the calculated predominance of dissolved forms:

(1') Co^{2+} / $HCoO_2^-$ (pH=10.57)

(2') Co^{2+} / Co^{3+} (E_0 =1.808)

(3') $HCoO_2^-$ / Co^{3+} (E_0= -0.065 + 0.1773 pH)

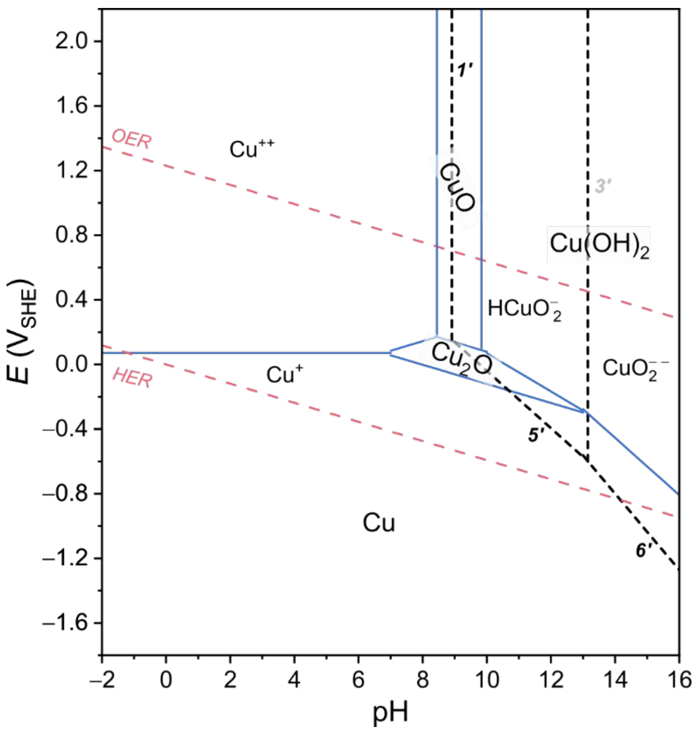

Figure S26. Pourbaix diagram of Cu constructed using experimental thermodynamic data[10] at 25 °C, assuming an aqueous ion concentration of 10^{-9} mol L^{-1}. The potential range used in this study corresponds to 0.073 V_{SHE} to −1.127 V_{SHE} at pH 12.3. The dashed gray lines outline the stability regions of the Cu species based on the calculated predominance of dissolved forms:

(1’) Cu^{2+} / $HCuO^{-}{}_{2}$ (pH = 8.91)

(3’) $HCuO^{-}{}_{2}$ /$HCuO^{2-}{}_{2}$ (pH = 13.15)

(5’) Cu^{+} / $HCuO^{-}{}_{2}$ (E_0 = 1.733 - 0.1773 pH)

(6’) Cu^{+} / $CuO^{2-}{}_{2}$ (E_0 = 2.510 - 0.2364 pH)

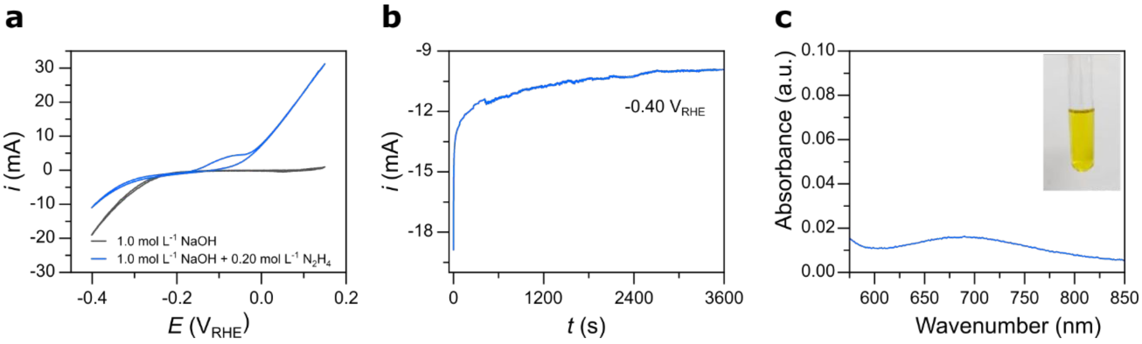

Figure S27. (a) Cyclic voltammogram of CA-reconstructed Co/Cu mixed oxide-derived electrocatalyst in 1.0 mol L^{-1} NaOH and 0.20 mol L^{-1} N_2H_4 from 0.15 V_{RHE} to −0.40 V_{RHE} at 1 mV s^{-1} scan rate; (b) chronoamperometric curve of hydrazine reduction at −0.40 V_{RHE}; and (c) post-electrolysis spectrophotometric detection of ammonia. Inset: photograph of the tested electrolyte.

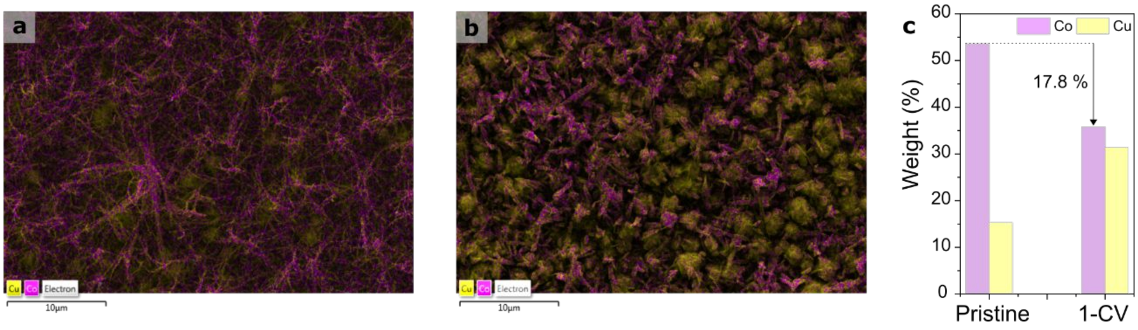

Figure S28. SEM/EDX Co and Cu elemental maps of (a) pristine and (b) after 1-CV in 0.02 mol L^{-1} NaOH from 0.80 V_{RHE} to −0.40 V_{RHE} at 2 mV s^{-1} scan rate.

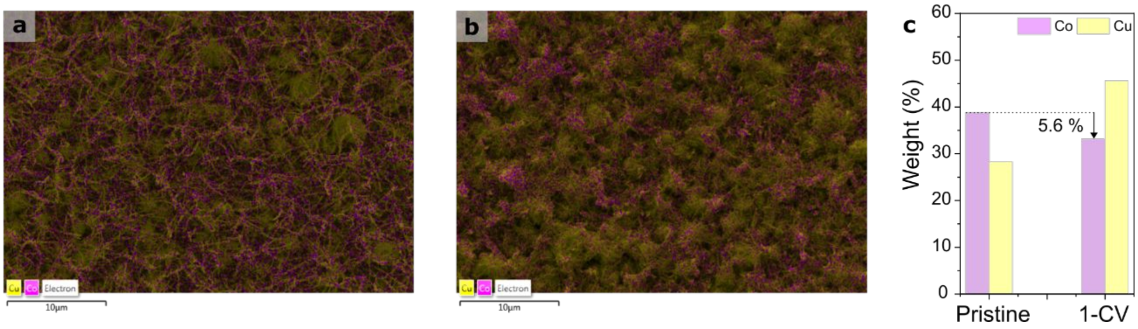

Figure S29. SEM/EDX Co and Cu elemental maps of (a) pristine and (b) after 1-CV in 0.02 mol L^{-1} NaOH + 0.02 mol L^{-1} $NaNO_3$ from 0.80 V_{RHE} to −0.40 V_{RHE} at 2 mV s^{-1} scan rate.

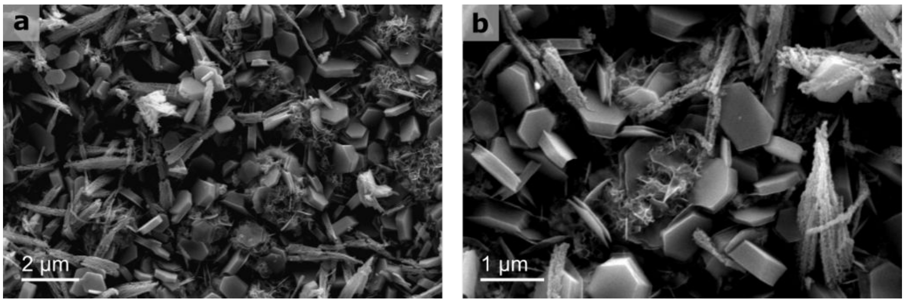

Figure S30. (a-b) SEM image of Co/Cu mixed-oxide electrocatalyst after 1-CV cycle in nitrate-containing electrolyte (0.02 mol L^{-1} NaOH + 0.02 mol L^{-1} $NaNO_3$) from 0.80 V_{RHE} to −0.40 V_{RHE} at 2 mV s^{-1} scan rate.

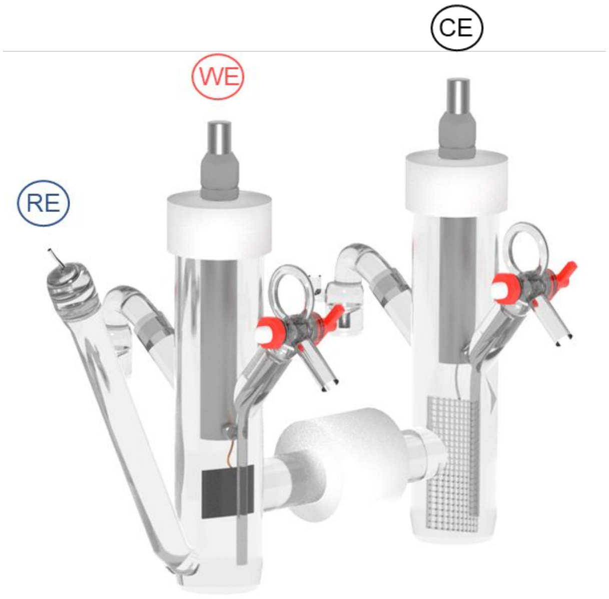

Figure S31. Custom-made H-type electrochemical used in the NO_3RR experiments. RE: reversible hydrogen electrode; WE: CA- and CV-reconstructed electrocatalyst; and CE: Pt mesh.

References

[1] D. Anastasiadou, Y. van Beek, W. Chen, T. Wissink, A. Parastaev, E. J. M. Hensen, M. Costa Figueiredo, *ChemCatChem* **2023**, *15*, e20220150.

[2] I. Messias, M. E. G. Winkler, G. F. Costa, T. Mariano, J. B. Souza Junior, I. T. Neckel, M. C. Figueiredo, N. Singh, R. Nagao, *ACS Appl Energy Mater* **2024**, *7*, 9034.

[3] L. Bai, F. Franco, J. Timoshenko, C. Rettenmaier, F. Scholten, H. S. Jeon, A. Yoon, M. Rüscher, A. Herzog, F. T. Haase, S. Kühl, S. W. Chee, A. Bergmann, R. C. Beatriz, *J Am Chem Soc* **2024**, *146*, 9665.

[4] S. Lu, G. Lin, H. Yan, Y. Li, T. Qi, Y. Li, S. Liang, L. Jiang, *ACS Catal* **2024**, *14*, 14887.

[5] W. He, J. Zhang, S. Dieckhöfer, S. Varhade, A. C. Brix, A. Lielpetere, S. Seisel, J. R. C. Junqueira, W. Schuhmann, *Nat Commun* **2022**, *13*, 1129.

[6] W. Yang, Z. Chang, X. Yu, P. Wu, R. Shen, L. Wang, X. Cui, J. Shi, *Advanced Science* **2025**, *12*, 2416386.

[7] Y. Shi, L. Chen, L. Xiong, X. Wang, Y. Yu, M. Yang, *Chemical Engineering Journal* **2025**, *507*, 160393.

[8] D. Li, S. Zhang, Z. Mao, M. Liu, K. Hu, D. Zhao, Z. Qv, L. Zhou, T. Shi, *RSC Adv* **2025**, *15*, 9461.

[9] W. Huang, W. Luo, J. Liu, B.-E. Jia, C. Lee, J. Dong, L. Yang, B. Liu, Q. Yan, *ACS Nano* **2024**, *18*, 20258.

[10] M. Pourbaix, in *Atlas of Electrochemical Equilibria in Aqueous Solutions*, National Association of Corrosion Engineers, **1974**, pp. 322–329.
